# Effects of Frozen Storage on Phospholipid Content in Atlantic Cod Fillets and the Influence on Diet-Induced Obesity in Mice

**DOI:** 10.3390/nu10060695

**Published:** 2018-05-30

**Authors:** Kristin Røen Fauske, Annette Bernhard, Even Fjære, Lene Secher Myrmel, Livar Frøyland, Karsten Kristiansen, Bjørn Liaset, Lise Madsen

**Affiliations:** 1 Institute of Marine Research, P.O. Box 7800, 5020 Bergen, Norway; kristin.fauske@hi.no (K.R.F.); annette.bernhard@hi.no (A.B.); even.fjaere@hi.no (E.F.); lenesecher.myrmel@hi.no (L.S.M.); livar.froyland@hi.no (L.F.); bjorn.liaset@hi.no (B.L.); 2 Department of Biomedicine, University of Bergen, 5020 Bergen, Norway; 3 Department of Biology, University of Copenhagen, 2100 Copenhagen, Denmark; kk@bio.ku.dk

**Keywords:** obesity, Western diet, cod, pork, free fatty acids, phospholipids, triacylglycerol, *n*-3 polyunsaturated fatty acids, mice

## Abstract

A large fraction of the *n*-3 polyunsaturated fatty acids (PUFAs) in cod fillet is present in the form of phospholipids (PLs). Freezing initiates hydrolysis of the PLs present in the fillet. Here, we compared the effects of Western diets based on frozen cod, fresh cod or pork with a diet based on casein in male C57BL/6J mice fed for 12 weeks at thermoneutrality. Diets based on fresh cod contained more PL-bound *n*-3 PUFAs (3.12 mg/g diet) than diets based on frozen cod (1.9 mg/g diet). Mice fed diets containing pork and fresh cod, but not frozen cod, gained more body and fat mass than casein-fed mice. Additionally, the bioavailability of *n*-3 PUFAs present in the cod fillets was not influenced by storage conditions. In a second experiment, diets with pork as the protein source were supplemented with *n*-3 PUFAs in the form of PL or triacylglycerol (TAG) to match the levels of the diet containing fresh cod. Adding PL-bound, but not TAG-bound, *n*-3 PUFAs, to the pork-based diet increased body and fat mass gain. Thus, supplementation with PL-bound *n*-3 PUFAs did not protect against, but rather promoted, obesity development in mice fed a pork-based diet.

## 1. Introduction

The changing of dietary patterns represents a tool to curb the development of obesity and type 2 diabetes [1]. Epidemiological studies have also indicated that the intake of dairy and plant-derived protein as well as protein from various seafood sources is associated with protection against obesity development, whereas a high intake of meat protein predicts higher weight gain [1,2]. In line with these findings, C57BL/6J mice fed a Western diet (40% of energy as fat, 44% of energy as carbohydrates and 16% of energy as protein) containing a mixture of lean seafood had lower adiposity than mice fed a Western diet containing a mixture of skinless chicken breast fillet, pork tenderloin and beef sirloin [3]. Furthermore, compared with low fat-fed mice, mice fed lean meat exhibited increased levels of fasting blood glucose, fasting plasma insulin and liver lipids. The energy intake was reduced by 8% when the mice were fed lean seafood, and this was further accompanied by a reduced feed efficiency. Exchanging meat from lean pork sirloins with cod fillets in diets with a similar composition of macronutrients led to attenuated obesity development accompanied with a 6% lower feed intake and a significantly reduced feed efficiency [4]. Further, the analysis of a second group of pork-fed mice that were pair-fed with the cod-fed mice corroborated the finding that cod, only in part, attenuates obesity development via a reduced feed intake [4].

In rat studies, the intake of both cod and soy proteins provided in a low-fat diet improved peripheral insulin sensitivity compared with rats fed a casein-based, low fat diet [5]. In a follow up study with high fat/high sucrose diets, it was demonstrated that in the absence of any effect on adipose tissue mass, feeding rats cod protein prevented the development of insulin resistance in skeletal muscle [6]. The diets in the latter study contained 67% energy as fat, 18% energy as sucrose and 15% energy as protein. 

Obesity development and energy intake in chicken and cod-fed mice were similar when C57BL/6J mice were fed chicken breast fillets or cod fillets in diets based on the same high fat/high sucrose diet [7] used by Lavigne et al. [6]. However, the intake of high fat/high protein diets with cod fillets as the protein source led to decreased obesity development compared with mice fed protein from chicken breast fillet or pork sirloin, but the cod-fed mice had greater adipose tissue mass than mice fed casein or soy as protein sources [8]. In the latter experiment, glucose tolerance and insulin sensitivity mirrored fat mass. Hence, data on the effect of cod intake on the development of obesity and regulation of glucose homeostasis has varied between experiments, possibly reflecting differences in specific experimental conditions.

In the abovementioned rat studies by Lavigne et al. [5,6], diethyl ether was used to remove the small amount of endogenous fat present in the cod fillets, whereas in our previous mice experiments, freeze-dried cod fillets were used [3,4,7,8]. Intake of a Western diet containing freeze-dried cod fillets led to increased accumulation of the marine *n*-3 polyunsaturated fatty acids (PUFAs), eicosapentaenoic acid (EPA) and docosahexaenoic acid (DHA) in both liver and red blood cells (RBCs) in mice [4]. A large fraction of the marine *n*-3 PUFAs in cod fillets is present in the form of phospholipids (PLs) [9], and it has been reported that the bioavailability as well as the anti-obesogenic and anti-steatotic effects of PL-bound *n*-3 PUFAs are superior to triacylglycerol (TAG)-bound *n*-3 PUFAs [10,11]. Hence, we have previously suggested that despite a low fat content, PL-bound *n*-3 PUFAs may mediate some dietary effects of cod [4].

If the dietary effects of cod are mediated via PL-bound *n*-3 PUFAs, the storage time of the fillets may be of importance. Cod has a short commercial catch season, spanning four months in the winter, and hence, freezing is a broadly used method for preservation to ensure the availability of cod throughout the low season [12]. It is known that frozen storage of cod fillets initiates enzymatic hydrolysis of the PLs present in the fillets [13,14], resulting in increased levels of free fatty acids (FFAs) in the frozen fillets with time [15,16]. In addition to a changed PL:FFA ratio, lipid oxidation and ice crystal formation have been shown to play important roles in protein denaturation and consequently, change the texture and quality of frozen cod fillets [17,18].

Considering the variable outcomes reported regarding the link between dietary cod intake and obesity development, and a possible role of cod freshness, i.e., the PL content of the cod, we here aimed to investigate whether frozen storage of cod influences the bioavailability of *n*-3 PUFAs as well as the ability of cod to modulate energy intake, development of obesity, and steatosis in obesity-prone C57BL/6J mice. We also investigated whether PL-bound *n*-3 PUFAs supplemented at a level comparable to that in fresh cod are able to affect the development of obesity and steatosis in mice fed Western diets.

## 2. Materials and Methods

### 2.1. Ethical Statement

The animal experiments were approved by the Norwegian Animal Research Authority (Norwegian Food Safety Authority; FOTS id.nr 7882). Animal care and handling were performed in accordance with national and international guidelines (Regulation on the use of animals in research, Ministry of Agriculture and Food, 1 July 2015; according to Directive 2010/63/EU of the European Parliament and of the Council of 22 September 2010).

### 2.2. Animal Studies

Two animal studies were performed. In both experiments, male C57BL/6J BomTac mice, 8 weeks of age at arrival, with an average body weight of 24.9 ± 0.1 g, average fat mass of 2.46 ± 0.06 g and average lean mass of 19.0 ± 0.1 g, were obtained from Taconic (Ejby, Denmark). The mice were housed (one animal per cage) in individually ventilated cages (IVC) in a thermoneutral environment (29 ± 1 °C) with 50% relative humidity and on a 12:12 h light-dark cycle. After one week of acclimatization to a low fat diet (based on the Ssniff EF R/M control diet (E15000-04), Soest, Germany), the mice were divided into feeding groups based on measurements of body weight and relative fat and lean mass. Five mice continued with the low fat diet to monitor normal weight and health development. The mice had free access to water and were fed their respective diets *ad libitum*. In both experiments, fresh water was provided twice per week, and fresh feed was provided three times per week. Body weight was recorded once per week and feed intake was recorded three times per week. In both experiments, the mice were sacrificed by cardiac puncture under isoflurane anesthesia (Isoba vet, Schering-Plough A/S, Farum, Denmark) after 12 weeks of feeding. Blood was collected from the heart into tubes containing EDTA. The RBC fraction was prepared by centrifugation (1500 G, 15 min, 4 °C) and stored at −80 °C until further analyses. Liver and adipose tissues were dissected out, weighed, snap-frozen in liquid nitrogen and stored at −80 °C until further analyses.

### 2.3. Lipid Class Composition

The lipid class composition of raw and heated fresh cod fillets, freeze dried powders, diets and mouse livers was measured using high-performance thin-layer chromatography (HPTLC), as described by Jordal et al. [19]. Lipids were extracted from the samples by adding 20× the amount of sample (*v*/*w*) of chloroform:methanol (2:1) with 0.01% BHT. After extraction of lipids, the samples were filtered, taken to dryness, and diluted in chloroform with 0.01% BHT, to obtain a final concentration of 5 mg/mL. One microliter of the solution was applied to a 20 × 10 cm HPTLC Silica 60 plate (Merck, Darmstadt, Germany) that had been pre-run in chloroform and activated at 110 °C for 30 min. The plates were developed to 48 mm in a polar solution of chloroform, isopropanol, methyl acetate, methanol and 0.25% (*w*/*v*) aqueous KCl (25:25:25:10:9, by volume) to separate polar from neutral lipid classes running at the solvent front. After drying, the plates were fully developed in isohexane, diethyl ether and acetic acid (80:20:1.5, by volume) to separate neutral lipids and cholesterol. Lipid classes were visualized by charring at 160 °C for 15 min after development in 3% copper acetate (*w*/*v*) in 8% (*v*/*v*) phosphoric acid for 10 s and identified by comparison with commercially available standards. Lipid classes were quantified using a densitometer (CAMAG TLC Scanner 3, CAMAG, Muttenz, Switzerland) and calculated using an integrator (winCATS Planar Chromatography Manager, Version 1.4.2, CAMAG, Muttenz, Switzerland). Finally, quantitative determination of lipid classes (mg lipid class/g tissue) was performed by establishing standard equations for each lipid class within a linear range of area, in addition to including a standard mixture of all the lipid classes at each HPTLC plate for corrections of between plate variations. The limit of quantification was 0.01 mg lipids/g sample. 

### 2.4. Fatty Acid Composition in the Polar and Neutral Lipid Fractions

The FA composition of RBCs and the FA composition in the polar and neutral lipid fractions of raw and heated fresh cod fillets, freeze-dried powders, diets and mouse livers were measured, as described by Liisberg et al. [4]. Lipids were extracted by adding 20× the amount of sample (*v*:*w*) of chloroform:methanol (2:1). After filtration of the extract, solvents were evaporated, and the residue was dissolved in 2% methanol in chloroform and separated into polar and neutral fractions using solid phase extraction (SPE). The SPE cartridge (Biotage Isolute SI 500 mg/10 mL, Uppsala, Sweden) was conditioned with 5 mL of hexane. The sample was then loaded and eluted with 10 mL 2% methanol in chloroform, and the neutral fraction was collected. Then, 15 mL of methanol was added, and the fraction was collected as the polar lipids. Methyl ester of C19:0 (nonadecanoic acid) was added to each fraction/sample as an internal standard, before saponifying the lipid samples with NaOH and methylating the FAs using 12% BF_3_ in methanol. The quantity of FAs in each fraction was determined by gas chromatography coupled with a flame ionization detector, identified by retention time using standard mixtures of methyl esters (Nu-Chek-Prep, Elysian, MN, USA) and quantified towards the internal standard under the conditions previously described by Torstensen et al. [20], based on Lie et al. [9]. The limit of quantification was 0.01 mg FA/g per sample. 

### 2.5. Experimental Diets

Western and low fat diets were prepared to match the macronutrient composition used in an earlier study [4]. We used casein powder (C8654 SIGMA, Merck, Darmstadt, Germany), fillets from fresh, wild-caught Atlantic cod (Lerøy Alfheim AS, Bergen, Norway) and fresh pork sirloins (H. Bragstad A/S, Bergen, Norway) as protein sources. Fresh cod fillets and pork sirloins were prepared as powders, as described by Liisberg et al. [4]. In all diets, the amounts of casein powder and pulverized freeze-dried cod fillets and pork sirloins added to achieve 200 g crude protein/kg diet were calculated from measurements of nitrogen content in the powders, determined by the Dumas method using a Leco FP 628 nitrogen analyzer (Leco Corporation Svenska AB, Täby, Sweden). The nitrogen to protein conversion factors used for the calculation of crude protein in the diets were N × 6.15 for casein and N × 5.6 for cod and pork [21]. Based on measurements of endogenous fat in the protein powders (described in the experiments), we balanced the diets with equal parts of margarine, lard and milk fat to achieve 180 g fat/kg diet. The diets were blended with a Crypto Peerless EF20 blender and analyzed for gross energy by bomb calorimetry (Parr Instrument, Moline, IL, USA).

Experiment 1: Half of a batch of raw fresh cod fillets and a whole batch of fresh pork sirloins were heated in a steamer to a core temperature of 70 °C, freeze-dried to >97% dryness, homogenized to powder and stored at −20 °C until use (12 weeks). The raw, fresh cod fillets were analyzed before and after heating to determine the lipid class composition (Table S1) and FA composition in the polar and neutral lipid fractions (Table S2). The remaining batch of raw, fresh cod fillets was stored at −20 °C to initiate enzymatic hydrolysis of the PLs, which ought to increase the FFA content in the raw cod fillets during freezing [13,14]. After 12 weeks of storage, the frozen cod fillets were thawed overnight at 4 °C, and prepared as powder, as described above. The lipid class compositions of the powders of frozen and fresh cod are shown in Table 1, and the FA compositions of the polar and neutral lipid fractions of the powders are shown in Table S3. The compositions of the diets are shown in Table S4, and the FA compositions of the polar and neutral lipid fractions in the frozen cod, fresh cod and pork-containing diets are shown in Table S5.

Experiment 2: Fillets of fresh cod and fresh pork sirloins were prepared as powders, as described above. The lipid class composition of the fresh cod powder is shown in Table S6, and the FA compositions of the polar and neutral lipid fractions are shown in Table S7. We aimed to investigate whether PL-bound EPA+DHA influenced obesity development and hepatic lipid accumulation in mice fed Western diets. Hence, the amount of EPA+DHA present in the fresh cod Western diet, 2.8 mg EPA+DHA/g diet (Table 2), was added to a Western diet containing pork. Equal parts of margarine, milk fat and lard were replaced with *n*-3 PUFA oils, either as PL-bound *n*-3 PUFAs extracted from herring roe diluted in soybean oil (pork *n*-3 PL diet), prepared by Innolipid AS (Aalesund, Norway), or TAG-bound *n*-3 PUFAs from cod liver oil (pork *n*-3 TAG diet) (Möller’s cod liver oil, Orkla Health, Oslo, Norway). The FA compositions of the polar and neutral lipid fractions of the oils are presented in Table S8. One Western diet containing pork was not supplemented with *n*-3 PUFAs (pork diet). The compositions of the diets are shown in Table S9, and the FA compositions of the polar and neutral lipid fractions of the Western diets are shown in Table 2.

### 2.6. Body Composition of the Mice

Free water, lean and fat mass were measured in live mice using a Bruker minispec LF50 Body composition Analyzer mq 7.5 (Bruker Optik GmBh, Ettlingen, Germany) as described by Halldorsdottir et al. [22]. 

### 2.7. Apparent Digestibility of Nitrogen and Fat

In both experiments, after six weeks of feeding, the mice were placed in cages with standard wood bedding for one week. All feces were collected from the cages, weighed and frozen at −20 °C until analysis. Nitrogen content in feces was determined with the Dumas method, as described above, and the total fat content in feces samples was determined gravimetrically after extraction with organic solvents before and after acidic hydrolysis, as described previously [7]. The apparent digestibilities of nitrogen and fat were calculated as follows: 100 × (intake (mg) − feces output (mg)/(intake (mg)).

### 2.8. Oral Glucose Tolerance Test

An oral glucose tolerance test (OGTT) was performed in Experiment 1 after 11 weeks of experimental feeding. Fasting blood glucose was measured in Experiment 2 after 10 weeks of experimental feeding. After 6 h of fasting, the mice were given 3 mg of glucose/g of lean mass by gavage. Blood was collected from the tail veins of conscious mice, and blood glucose was measured using a glucometer (Contour^®^ Next, Ascensia Diabetes care Holdings AG, Basel, Switzerland). Blood glucose was measured in the fasting state (0), and again at 15, 30, 60 and 120 min after the glucose injection [23]. Plasma was collected in the fasting state (0), and again after 15, 30, and 120 min following the glucose injection. An Ultra Sensitive Mouse Insulin ELISA Kit (Crystal chem (Europe) catalog# 90080, Zaandam, The Netherlands) was used according to the producer’s manual to quantify the plasma insulin collected during the OGTT. The incremental area under the curve (iAUC) for the OGTT was calculated using the fomula iAUC = AUC − (basal glucose × 120 min).

### 2.9. Statistics

Mice fed a low fat diet were only used as a reference for normal weight and health development, and thus, were not included in the statistical analyses. All data are presented as means ± SEMs. The homogeneity of variance in the data was established by Bartlett’s test, and the data were compared between groups using one-way ANOVA followed by Fisher’s multiple comparison post hoc tests. In Experiment 1, Western diet groups were compared with a reference group fed a casein-based Western diet. The reference group was not included in the bioavailability measurements of FAs in liver lipids and RBCs; hence, data were compared between the experimental Western diet groups. In Experiment 2, data were compared between all groups of Western diet-fed mice. In both experiments, cumulative energy intake was analyzed by repeated measures ANOVA and Fisher’s LSD multiple comparison post hoc tests. Group means were considered to be statistically different at *p* < 0.05. Statistical analyses were performed using Graph Pad Prism version 7.01 (GraphPad software, La Jolla, CA, USA).

## 3. Results 

### 3.1. Frozen Storage of Cod Fillets Decreased the Polar Lipid:FFA Ratio

Raw fresh cod fillets contained 5.7 mg lipids/g (Table S1). Heating the fillets in a steamer to a core temperature of 70 °C led to a loss of water and FFAs, and the heated fillets contained 10.5 mg lipids/g. The relative levels of FFAs were reduced from 12.7 to 6.3% upon heating, but the relative amounts of either polar or neutral lipids did not change (Table S1). The contents of EPA and DHA in the polar and neutral lipid fractions were 55.4% and 49.2%, respectively, in the raw fillets, and heating did not significantly influence the EPA and DHA contents (Table S2). As expected, the relative amounts of FFAs were higher in freeze-dried frozen cod fillets than in freeze-dried fresh fillets, whereas the relative polar lipid levels were lower (Table 1). Consequently, storage of the cod fillets prior to heating and freeze-drying decreased the polar lipid:FFA ratio (Table 1). The relative proportions of EPA and DHA in both the polar and the neutral lipid fractions were comparable in powders from freeze-dried fresh and frozen cod, respectively (Table S3).

The energy contents in the diets (Table S4) were confirmed using bomb calorimetry and were 20.38 ± 0.06 kJ/g in the Western diets, and 18.51 ± 0.03 kJ/g in the low fat reference diet. FFAs at the level above the limit of quantification were only present in the Western diet containing frozen cod (1.6 mg FFAs/g diet). We determined the contribution of endogenous fat from the protein sources in the diets to the FA composition in both the polar and neutral lipid fractions extracted from the diets (Table S5). Due to the dominant contribution from the added milk fat, margarine, and lard, the most pronounced differences between the diets were observed in the polar fractions. Still, the contents of the marine *n*-3 PUFAs, EPA and DHA were higher in both fractions extracted from the diets containing cod compared to the pork-containing diet. Due to the relatively high levels of *n*-3 PUFA alpha-linolenic acid (ALA) in the neutral lipid fractions extracted from all Western diets, the difference in the *n*-6:*n*-3 ratio between the pork and cod diets was far more pronounced in the polar lipid fraction than in the neutral fraction. Still, the neutral lipid fraction of the frozen cod-based diet also contained higher amounts of total EPA and DHA compared to the fresh cod-based diet: 1.2% versus 0.52%. However, in the polar lipid fraction, the sum of EPA and DHA represented as much as 39% and 45% of the total identified FAs from diets containing frozen and fresh cod, respectively.

### 3.2. Fresh Cod, But Not Frozen Cod, Is More Obesogenic than Casein in a Western Diet

**Figure 1:**
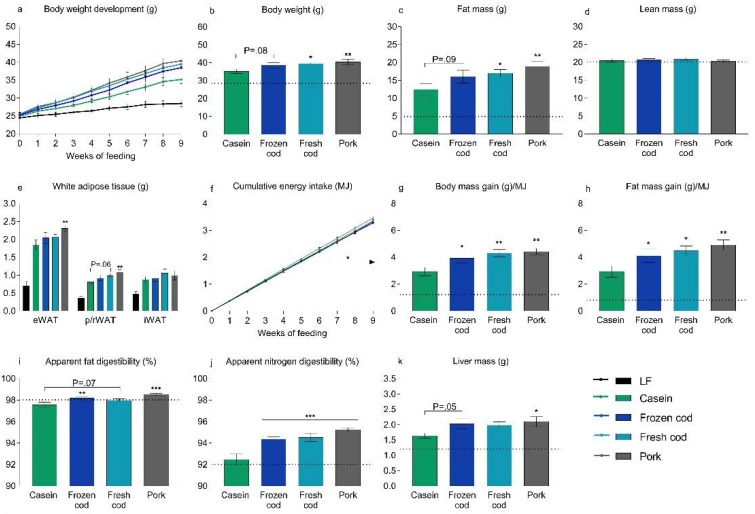
Effects of Western diets with different protein sources on body composition, energy intake and tissue weights. Male C57BL/6J mice were fed Western diets containing casein, frozen cod, fresh cod or pork as protein sources for 12 weeks. As a reference, a group of low fat (LF)-fed mice (*n* = 5) was also included and is shown as a dotted line. (**a**) Body weight development was measured and is shown for the first 9 weeks of feeding. (**b**) Body weight was measured and (**c**) fat mass and (**d**) lean mass were determined using nuclear magnetic resonance after 9 weeks of feeding. (**e**) Epididymal white adipose tissue (eWAT), inguinal white adipose tissue (iWAT) and perirenal/retroperitoneal white adipose tissue (p/r WAT) were dissected out after 12 weeks of feeding, and their masses were recorded; (**f**) Feed intake was recorded continuously, and cumulative energy intake (MJ) was determined and is shown for the first 9 weeks of feeding. The arrow indicates a significantly (*p* < 0.05) decreased energy intake in mice fed frozen cod and fresh cod compared to mice fed casein. (**g**) After 9 weeks of feeding, body mass gained per energy unit consumed and (**h**) fat mass gained per energy unit consumed were calculated; Apparent (**i**) fat and (**j**) nitrogen digestibility (%) were calculated based on feed intake and feces collected in the 6th week of feeding. (**k**) Livers were dissected out and weighed after 12 weeks of feeding. Data are presented as means ± SEMs (*n* = 10) and were analyzed using one-way ANOVA followed by Fisher’s LSD post hoc tests. Cumulative energy intake was analyzed by repeated measures ANOVA and Fisher’s LSD post hoc tests. *, ** and *** represent significant different from the Western diet containing casein at *p* < 0.05, *p* < 0.01 and *p* < 0.001 levels, respectively.

To investigate if prolonged storage of fresh cod at −20 °C influences the ability of cod to attenuate obesity and steatosis development, C57BL/6J mice were fed Western diets containing frozen or fresh cod for 12 weeks. For comparison, mice were fed a low fat diet or a Western diet using casein as the protein source. Compared with mice fed a casein-based Western diet, mice fed pork and fresh cod, but not frozen cod, gained significantly more weight after 9 weeks (Figure 1a,b). Similarly, compared with mice fed casein, fat mass was higher in mice fed pork and fresh cod, but not frozen cod (Figure 1c), while lean body mass was comparable in mice fed either of the diets (Figure 1d). After 12 weeks, epididymal white adipose tissue (eWAT) mass was significantly higher in pork-fed mice, but not in frozen or fresh cod fed mice when compared to mice fed casein (Figure 1e). Of note, the mass of the perirenal/retroperitoneal white adipose tissue (p/r WAT) depot in mice fed fresh cod, but not frozen cod, was borderline (*p* = 0.057) higher than that of casein-fed mice (Figure 1e). Mice fed frozen cod, fresh cod, and pork were comparable to casein-fed mice in relation to the inguinal white adipose tissue mass (iWAT; Figure 1e).

After 6 weeks of feeding, a reduced energy intake was observed in mice fed frozen and fresh cod, but not in pork-fed mice, compared to casein-fed mice (Figure 1f). Compared with casein-fed mice, body mass gained per unit of energy intake and fat mass gained per unit of energy intake were higher in mice fed frozen cod, fresh cod and pork (Figure 1g,h). The apparent digestibility of fat was significantly higher in mice fed frozen cod and pork and tended (*p* = 0.07) to be higher in mice fed fresh cod compared to casein-fed mice (Figure 1i). The apparent digestibility of nitrogen was significantly higher in all mice fed cod and pork compared to casein-fed mice (Figure 1j). 

The liver mass was significantly higher (22%; *p* < 0.05) in pork-fed mice compared to casein-fed mice. The liver mass was borderline (*p* = 0.05) higher in mice fed frozen cod compared to casein-fed mice, while the liver mass of fresh cod fed mice was not statistically different from casein-fed mice (Figure 1k). No differences in glucose tolerance, fasting plasma insulin or fasting blood glucose were observed among groups (Figure S1). Hence, Western diets containing fresh cod, but not frozen cod, appear to be more obesogenic than a Western diet based on casein as the protein source. 

### 3.3. Bioavailability of n-*3* PUFAs in Frozen and Fresh Cod Diets

**Figure 2:**
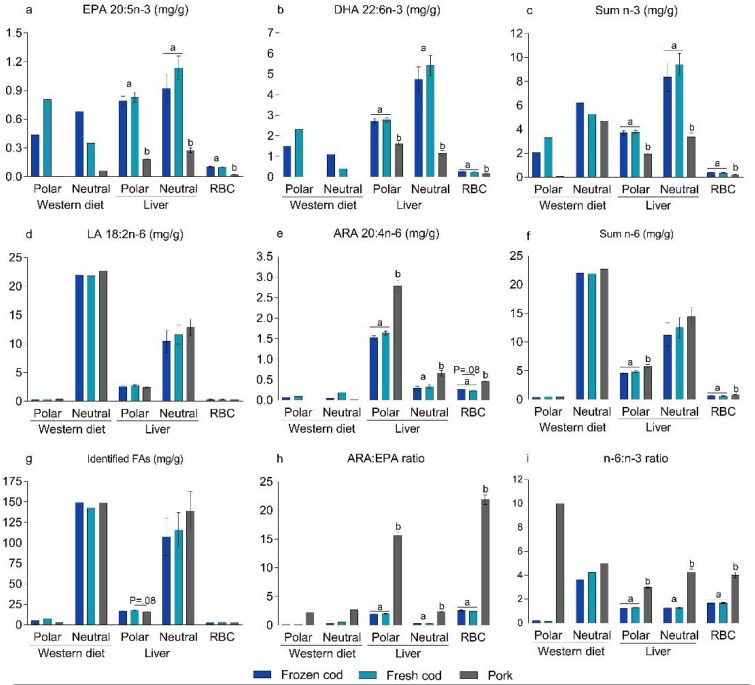
Fatty acid (FA) composition in polar and neutral lipid fractions in Western diets based on frozen cod, fresh cod, and pork as protein sources, and in mouse livers and red blood cells (RBCs) following intake of the different diets. Data from the Western diets represent the means of three samples, and data from the livers and RBCs represent means ± SEMs (*n* = 10). Results indicate mg FAs in the polar and neutral lipid fractions/g Western diet and livers, and mg FAs/g RBCs. Lipids from Western diets and livers were extracted and separated into polar and neutral lipid fractions. The FA compositions of the fractions from the Western diets and livers as well as the FA compositions of extracted lipids from RBC were quantification and determined; (**a**) eicosapentaenoic acid (EPA); (**b**) docosahexaenoic acid (DHA); (**c**) sum *n*-3; (**d**) linoleic acid (LA); (**e**) arachidonic acid (ARA); (**f**) sum *n*-6; (**g**) identified fatty acids (FAs); (**h**) arachidonic acid: eicosapentaenoic acid ratio (ARA:EPA); (**i**) *n*-6:*n*-3 ratio. Data from livers and RBCs were analyzed using one-way ANOVA followed by Fisher’s LSD post hoc tests. Different letters denote statistical significance (*p* ≤ 0.05) between the groups.

To investigate if the bioavailability of EPA and DHA was influenced by frozen storage of the cod fillets, we measured the FA composition in RBCs and livers collected from the mice. We found that the EPA and DHA contents of the frozen and fresh cod containing diets were similar in the Western diets (3.7 mg/g EPA+DHA vs. 3.87 mg/g EPA+DHA, respectively), but distributed differently between the polar and neutral lipid fractions. The frozen cod diet contained 0.44 mg/g EPA and 1.49 mg/g DHA in the polar lipid fraction and 0.68 mg/g EPA and 1.10 mg/g DHA in the neutral lipid fraction (Figure 2a,b and Table S5). In comparison, the fresh cod diet contained 0.81 mg/g EPA and 2.32 mg/g DHA in the polar lipid fraction and 0.35 mg/g EPA and 0.40 mg/g DHA were present in the neutral lipid fraction (Figure 2a,b and Table S5). Hence, when raw fresh cod fillets were stored at −20 °C for 12 weeks prior to preparation of powders, lower proportions of EPA and DHA were present in the polar lipid fraction and higher proportions were found in the neutral lipid fraction compared to the diet prepared using fresh cod.

Compared to pork-fed mice, mice fed frozen and fresh cod had significantly higher levels of EPA, DHA, and sum *n*-3, and lower levels of ARA in hepatic polar and neutral lipids and in RBCs (Figure 2a–c,e and Tables S10 and S11). However, the levels of EPA, DHA, sum *n*-3, and ARA were similar in RBCs and liver lipids of mice fed frozen cod and fresh cod, suggesting that the bioavailability of *n*-3 PUFAs present in the cod fillets was not influenced by the storage conditions. In the present study, despite a higher content of PL-bound *n*-3 PUFAs, a Western diet containing fresh cod, but not frozen cod, appeared to be more obesogenic than a casein-based Western diet, suggesting that PL-bound *n*-3 PUFAs did not mediate an anti-obesogenic effect. To the contrary, our results indicate that the PL-bound *n*-3 PUFAs present in a Western diet may rather increase obesity development.

### 3.4. Supplementation with n-*3* PUFAs in Pork-Based Western Diets

To further investigate the effect of PL-bound *n*-3 PUFAs in a Western diet on energy intake, obesity development, and hepatic steatosis, new fresh cod fillets were obtained. After heating and freeze-drying, the fresh cod fillet powder contained 39 mg lipids/g powder, and the relative levels of polar lipids and FFA were 94% and 1%, respectively (Table S6). The freeze dried fresh cod powder was blended into a Western diet. The polar lipid fraction of the diet contained 2.47 mg EPA+DHA/g diet (Table 2). To investigate whether PL-bound *n*-3 PUFAs increase obesity development in mice fed Western diets, we also prepared diets using pork, with or without addition of the same amount of PL-bound *n*-3 PUFAs as found in the fresh cod diet. The pork-containing diet supplemented with PL-bound *n*-3 PUFAs (pork *n*-3 PL diet) contained 2.7 mg EPA+DHA/g diet in the polar lipid fraction. The EPA:DHA ratios were similar in the fresh cod diet (0.39) and the pork *n*-3 PL diet (0.38) (Table 2). 

We also supplemented a pork diet with TAG-bound *n*-3 PUFAs (pork *n*-3 TAG diet), with an EPA:DHA ratio similar to the pork *n*-3 PL diet, as a control for the bioavailability of PL-bound EPA+DHA. As we were unable to obtain TAG-bound *n*-3 PUFAs with an EPA:DHA ratio similar to cod fillets and PL-bound *n*-3 PUFAs, the EPA:DHA ratio was somewhat higher in the pork *n*-3 TAG diet (0.725) than in the other diets. However, FA analyses demonstrated that the pork *n*-3 TAG diet contained 2.8 mg/g EPA+DHA in the neutral lipid fraction (Table 2), and EPA+DHA levels were hence comparable. 

### 3.5. Adding PL-Bound n-*3* PUFAs to a Western Diet Containing Pork Promoted Obesity in Mice 

**Figure 3:**
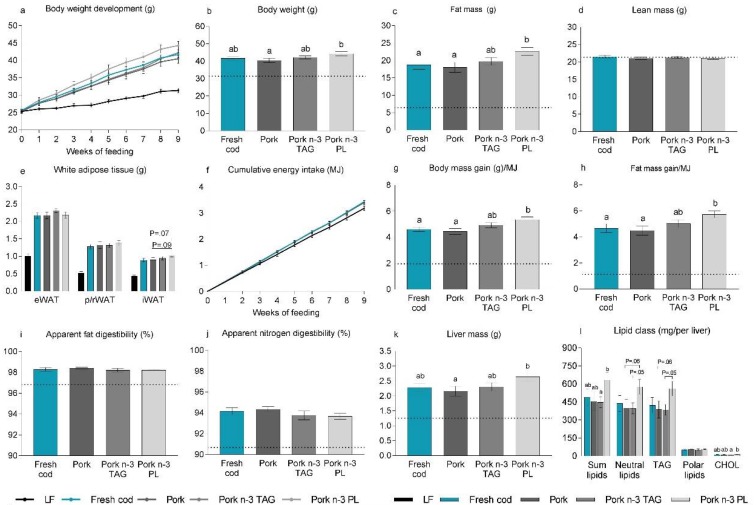
Effects of pork-containing Western diets with added *n*-3 PUFAs to match the level of *n*-3 PUFAs in a Western diet containing fresh cod. Male C57BL/6J mice were fed Western diets containing either fresh cod or fresh pork as a protein source for 12 weeks. The two pork diets were supplemented with either TAG-bound (pork *n*-3 TAG) or PL-bound (pork *n*-3 PL) EPA+DHA at levels matching the content of the fresh cod-containing diet. As reference, a group of low fat (LF)-fed mice (*n* = 5) was included and is shown as a dotted line. (**a**) Body weight development was determined and is shown for the first 9 weeks of feeding. (**b**) Body weight was measured and (**c**) fat and (**d**) lean mass were determined using nuclear magnetic resonance after 9 weeks of feeding. (**e**) Epididymal white adipose tissues (eWAT), inguinal white adipose tissues (iWAT) and perirenal/retroperitoneal white adipose tissues (p/r WAT) were dissected out after 12 weeks of feeding and their masses were recorded. (**f**) Feed intake was recorded continuously, and cumulative energy intake (MJ) was determined and is shown for the first 9 weeks of feeding; (**g**) After 9 weeks of feeding, the amount of body mass gained per energy unit consumed and (**h**) fat mass gained per energy unit consumed were calculated; Apparent (**i**) fat and (**j**) nitrogen digestibilities (%) were calculated based on feed intake and feces collected during the 6th week of feeding; (**k**) After 12 weeks of feeding, livers were dissected out and weighed (**l**). Lipids were extracted from livers (*n* = 7) and total lipids, neutral lipids, triacylglycerol (TAG), polar lipids and cholesterol (CHOL) were quantified, and results are presents as mg lipids per liver (mg lipids/g liver × liver weight (g)). Data are presented as means ± SEMs (*n* = 14–15), except for the lipid class analysis (*n* = 7) and were analyzed using one-way ANOVA followed by Fisher’s LSD post hoc tests. Cumulative energy intake was analyzed by repeated measures ANOVA and Fisher’s LSD post hoc tests. Different letters denote statistical significance (*p* ≤ 0.05) between the groups.

The Western diets containing fresh cod or pork supplemented with either TAG or PL-bound *n*-3 PUFAs containing similar amounts of *n*-3 PUFAs and a Western diet containing pork without supplementation were fed to C57Bl/6 mice for 12 weeks. After 9 weeks of feeding, intake of the pork diet with the PL-bound, but not TAG-bound, *n*-3 PUFAs resulted in a higher average body weight and fat mass compared to mice fed pork without *n*-3 PUFAs (Figure 3a–c). Fresh cod and unsupplemented pork-fed mice did not differ in body weight or fat mass (Figure 3a–c). Mice fed the pork *n*-3 PL diet tended to have a higher average iWAT mass compared to fresh cod-fed mice (*p* = 0.07) and pork-fed mice (*p* = 0.09) (Figure 3e). However, there were no significant differences in the white adipose tissue mass of mice fed any of the Western diets (Figure 3e). After 9 weeks of feeding, lean mass and energy intake were similar between all Western diet-fed mice (Figure 3d,f). 

Calculating body and fat mass gain relative to energy intake after 9 weeks of feeding revealed that mice fed the pork *n*-3 PL diet gained significantly more body and fat mass per unit of energy intake compared to intake of both the fresh cod- and pork-containing diets (Figure 3g,h). Mice fed pork supplemented with TAG-bound EPA+DHA had in between values. However, the increased body and fat mass gain per unit of energy intake did not appear to be caused by higher fat or nitrogen digestibility in mice fed the pork *n*-3 PL diet (Figure 3i,j). Furthermore, compared to mice fed the pork diet with no supplementation of EPA+DHA, the intake of PL-bound *n*-3 PUFAs, but not TAG-bound *n*-3 PUFAs, led to increased liver mass (Figure 3k) and increased hepatic accumulation of lipids and cholesterol in the liver (Figure 3l). Additionally, neutral lipids and TAGs were borderline higher in the livers of pork *n*-3 PL-fed mice than pork *n*-3 TAG-fed mice (*p* = 0.05) and pork *n*-3 TAG-fed mice (*p* = 0.06). Hepatic lipid accumulation was similar between fresh cod, pork and pork *n*-3 TAG-fed mice. No differences in fasting blood glucose were observed between groups (Figure S2). Hence, supplementation of PL-bound *n*-3 PUFAs to a pork diet did not protect against, but rather promoted, obesity and hepatic lipid accumulation in mice fed Western diets. 

### 3.6. Bioavailability of PL-Bound n-3 PUFAs

To evaluate the bioavailability of the dietary *n*-3 PUFAs, EPA and DHA, we analyzed the FA compositions of RBCs collected from the mice. There were no differences in the levels of EPA+DHA in RBCs collected from mice fed fresh cod, pork *n*-3 PL or pork *n*-3 TAG diets (Table 3). The ARA:EPA ratio was significantly higher in pork *n*-3 PL-fed mice compared to pork *n*-3 TAG and fresh cod-fed mice. Supplementation of *n*-3 PUFA bound to TAG or PL had the same ability to increase the levels of EPA+DHA and to decrease the ratio of *n*-6:*n*-3 PUFAs in RBCs. Hence, the bioavailability of TAG and PL-bound *n*-3 PUFAs supplemented to a pork-based diet were similar. Further, pork-fed mice had the highest *n*-6:*n*-3 ratio in their RBCs and additionally, had lower body weights and fat and liver masses compared to mice fed pork supplemented with *n*-3 PUFAs as TAG or PL. Thus, a decreased *n*-6:*n*-3 ratio in RBCs did not protect against obesity. Hence, we conclude that supplementation of a pork-based diet with *n*-3 PUFAs at relatively low levels did not protect against obesity, but rather increased the development of diet-induced obesity.

## 4. Discussion

The present study in mice provides surprising evidence that frozen storage of cod modulates the ability of cod intake to attenuate obesity development when incorporated into a Western diet. Cod is a lean fish, but marine *n*-3 PUFAs comprise a large fraction of the FAs in muscle PLs. It has been reported that the bioavailability as well as the anti-obesogenic and anti-steatotic effects of PL-bound *n*-3 PUFAs are superior to those of TAG-bound *n*-3 PUFAs [11]. Previously, we showed that in comparison to pork-fed mice, mice fed frozen cod exhibit diminished development of obesity and steatosis, accompanied with a lower *n*-6 to *n*-3 ratio in RBC and liver PLs as well as lower circulating levels of the two major ARA-derived endocannabinoids: *N*-arachidonoylethanolamine (AEA) and 2-arachidonoylglycerol (2-AG) [4]. Hence, we suggested that the PL-bound *n*-3 PUFAs may, at least in part, mediate the effects of a cod-containing diet on metabolism [4]. 

The levels of PLs are higher in fresh, than in stored, frozen cod fillets, as frozen storage leads to enzymatic hydrolysis of PLs [13,14], and accordingly, we expected that the intake of fresh cod fillets would attenuate obesity development more efficiently than the intake of cod fillets that had been stored at −20 °C. As anticipated, the levels of PLs decreased during frozen storage, and hence, although diets prepared with frozen cod contained similar total amounts of EPA+DHA to diets prepared from fresh cod, a lower proportion of *n*-3 PUFAs was present in the polar lipid fraction in the diets prepared with frozen cod. However, this did not translate into lower bioavailability. Further, mice fed a Western diet containing fresh cod, but not frozen cod, gained more fat mass than casein-fed mice. This indicates that a high proportion of EPA+DHA in PLs accentuates, rather than attenuates, obesity. In line with this, we observed that supplementing a pork-containing diet with the same amount of *n*-3 PUFAs present in fresh cod in the form of PLs, but not as TAGs, increased weight gain and fat mass in mice after 9 weeks. 

In contrasting to our results, previous studies have reported a superior effect of *n*-3 PUFAs in the form of PL. Some [24,25,26,27], but not all [11,28] used krill oil as the single source for PL-bound *n*-3 PUFAs. Krill oil is extracted from Antarctic krill (*Euphausia superba*), where 30–65% of the FAs are incorporated into PLs, mainly phosphatidylcholine [29]. Also, krill oil contains choline, which is a conditionally essential nutrient, and is also important for the transport of lipids [30]. Moreover, krill oil contains vitamin E, which may protect the unsaturated bounds in PUFAs from oxidation and the biological membranes from lipid peroxidation [31], as well as astaxanthin, a powerful antioxidant, which has been linked to the prevention and reversion of diet-induced insulin resistance and steatohepatitis in mice [32]. While the EPA:DHA ratio in krill oil is 1.8 [33], the EPA:DHA ratio in the polar fraction of the freeze-dried fresh cod has a ratio of 0.34 (Table S3), and PLs extracted from herring roe have a EPA:DHA ratio of 0.38 (Table S8). EPA enriched PLs have been demonstrated to attenuate high fat diet-induced obesity and hyperlipidemia more efficiently than DHA-enriched PLs [34]. Additionally, EPA and DHA are metabolized differently in rat livers [35]. A major functional difference between EPA and DHA relates to their conversion to different eicosanoids, including pro-resolving mediators that may play important roles in the development of different metabolic disorders [36]. Furthermore, it has been demonstrated that EPA is the hypotriacylglycerolemic component of fish oil [37]. Hence, our study cannot be directly compared with studies using krill oil.

Using PLs from marine fish, Rossmeisl et al. [11] demonstrated that the superior anti-obesogenic effect of PL-bound, compared to TAG-bound, *n*-3 PUFAs was related to higher bioavailability [11]. In agreement with this, earlier studies from our laboratory have demonstrated that increased *n*-3:*n*-6 ratios in fish fillets and in PLs from livers and RBCs collected from mice consuming fish-containing diets, are associated with reduced obesity [4,38,39,40]. However, in the present study, the dietary *n*-3 PL-bound PUFAs were not incorporated into mice RBCs to a higher degree than TAG-bound *n*-3 PUFAs. Of note, in line with our results, a critical review published in 2014 concluded that there was no evidence for greater bioavailability of *n*-3 PUFAs from PLs compared with TAGs [41]. Surprisingly, we did not observe an anti-obesogenic effect of PL-bound *n*-3 PUFAs, but rather, a weak obesity promoting effect, which may relate to the housing temperature and the use of pork-based diets. 

To our knowledge, we are the first to investigate how PL-bound *n*-3 PUFAs affect the obesity phenotype of mice fed Western diets using a pork-based background diet in a thermoneutral environment. Housing mice under thermoneutrality is claimed to be an advantageous step towards aligning murine energy metabolism to human energy metabolism [42], but possible effects of *n*-3 PUFAs on energy expenditure in brown or brown-like adipocytes [43,44] may be masked. At ambient temperatures below 30 °C, higher expression of the uncoupling protein 1 (UCP1) allows energy to be dissipated in the form of heat, and its expression is positively correlated with metabolic inefficiency and low energy efficiency [45]. UCP1-KO as well as fatty acid elongase-2 (Elovl2)-KO mice require housing under thermoneutrality to reveal the obesity phenotype [46,47]. Cold-exposure and even ambient housing temperatures of 20–22 °C require higher food intakes to meet the increased energy demand for thermogenesis [42,45], and a reduction in the ambient temperature has been demonstrated to attenuate high fat diet-induced obesity [48]. Performing experiments at a temperature at which the capacity for thermogenesis is reduced may have contributed to the increased obesity development in the present study. Along this line, it is also important to note that in the experiments in which an anti-obesogenic effect of PL-bound *n*-3 PUFA was observed, the background diets were based on casein [11,49]. 

We have previously reported that, at least in mice fed high protein/high fat diets, a characteristic brown adipocyte morphology of interscapular brown adipose tissue is dependent on the protein source [8]. Casein stands out as the most efficient protein source for preserving the brown adipocyte morphology, whereas the intake of proteins from pork and chicken leads to a more white-like adipocyte morphology. Hence, the thermogenic capacity may be further reduced by using proteins from pork instead of casein in the background diet. Therefore, we cannot exclude the possibility that both the housing temperature and the use of pork protein may have contributed to the unexpected obesogenic effect of PL-bound *n*-3 PUFAs. 

Results from an earlier study showed that mice fed frozen cod in a Western diet had 6% lower energy intakes compared to mice fed pork *ad libitum,* and additionally, mice fed frozen cod were leaner than pork-fed mice [4]. Compared to mice fed casein in the first experiment, we observed lower energy intakes in both frozen and fresh cod-fed mice, but not pork-fed mice after 6 weeks of feeding. In the second experiment, mice fed fresh cod and pork were compared, and we observed no differences in energy intake or diet-induced obesity between the mice. The freeze-dried fresh cod powder in the first experiment was stored at −20 °C for 12 weeks prior to be blended into a Western diet. Although the fresh cod powder was freeze-dried to >97% dryness before storage, we cannot rule out that the 12 weeks storage may have affected the powder. In contrast, the fresh cod powder in the second experiment was blended directly into the diet after preparation with minimum storage at −20 °C and was fed to the mice. It has been shown that storage of cod fillets at −23 °C leads to increased development of off flavors, affecting the quality of cod fillets [50]. The freezing of cod fillets initiates deterioration in flavor through rancidity, an undesirable fishy taste and other off flavors, believed to reflect the formation of low-molecular weight compounds from lipid oxidation and protein denaturation [51]. Hence, we cannot exclude the possibility that differences in the frozen storage time led to the observed differences in energy intake. To investigate this possibility, feeding mice diets containing cod frozen for longer periods to systematically increase the FFA:PL ratio of lipids would be required.

An important limitation in the study design is that we were unable to obtain *n*-3 PUFAs extracted from cod fillets, but used PL-oil extracted from herring roe. Still, the EPA:DHA ratio was similar to that of the fresh cod powder, and we supplemented the pork-containing diet with the same amount of *n*-3 PUFAs as found in the fresh cod diet. Another limitation is that we did not analyze other compounds that may have affected the quality of the frozen cod fillets. It would be of interest to investigate whether the accumulation of compounds from protein denaturation and lipid oxidation products during frozen storage is related to diet-induced obesity and energy intake in mice fed frozen cod fillets. It would further be of interest to investigate how PL-bound *n*-3 PUFAs vs. TAG-bound *n*-3 PUFAs affect obesity development when combined with other protein sources than pork.

In conclusion, compared with mice fed a casein-based Western diet, mice fed fresh cod, but not frozen cod with a reduced PL content, showed increased obesity development. Furthermore, the intake of a pork-containing diet with PL-bound, but not TAG-bound *n*-3 PUFAs, led to significantly average higher body weight and fat mass compared to the intake of a pork-based diet that was not supplemented with *n*-3 PUFAs. The present study points to a possible surprising effect of frozen storage of cod in relation to metabolism and obesity development and raises an interesting question as to whether similar effects of intake of fresh cod versus frozen cod may be observed in humans.

## Figures and Tables

**Table 1:** Lipid class composition in freeze-dried fresh and frozen cod fillets.

	Freeze-Dried Frozen Cod Fillets		Freeze-Dried Fresh Cod Fillets	
Lipid Class	mg/g	%	mg/g	%
PC	18.80 ± 0.3	42.0	36.5 ± 0.8	57.9
PE	5.1 ± 2.2	11.5	14.2 ± 0.3	22.6
PI	<0.01	<0.01	0.48 ± 0.05	0.76
PS	0.12 ± 0.02	0.27	0.86 ± 0.07	1.4
LPC	2.43 ± 0.03	5.43	2.4 ± 0.1	3.9
SM	0.66 ± 0.02	1.48	0.81 ± 0.03	1.28
CL	0.145 ± 0.005	0.33	0.64 ± 0.06	1.0
Sum polar lipids	27.3 ± 0.5	61.0	56.0 ± 0.7	88.8
FFA	15.1 ± 0.2	33.72	4.30 ± 0.07	6.8
CHOL	2.36 ± 0.03	5.27	2.64 ± 0.04	4.20
TAG	<0.01	<0.01	0.11 ± 0.03	0.18
DAG	<0.01	<0.01	<0.01	<0.01
CE	<0.01	<0.01	<0.01	<0.01
Sum neutral lipids	17.4 ± 0.2	39.0	7.1 ± 0.1	11.2
Sum lipids	44.7 ± 0.7		63.0 ± 0.7	
Polar lipid:FFA ratio	1.809 ± 0.008		13.0 ± 0.2	

Results are presented as means ± SEMs of three samples and indicate mg lipids/g and percent lipid class of total lipids in the freeze-dried frozen and fresh cod fillets. Abbreviations: PC, phosphatidylcholine; PE, phosphatidylethanolamine; PI, phosphatidylinositol; PS, phosphatidylserine; LPC, lysophosphatidylcholine; SM, sphingomyelin; CL, cardiolipin; FFA, free fatty acids; CHOL, cholesterol; TAG, triacylglycerol; DAG, diacylglycerol; CE, cholesteryl ester.

**Table 2:** Fatty acid composition in the polar and neutral lipid fractions isolated from Western diets.

Fatty Acid (mg/g)	Fresh Cod	Pork	Pork *n*-3 TAG	Pork *n*-3 PL
Polar lipid fraction				
Sum SFA	1.20 ± 0.02	0.76 ± 0.01	1.64 ± 0.07	2.12 ± 0.08
Sum MUFA	0.73 ± 0.03	0.474 ± 0.007	1.20 ± 0.06	1.014 ± 0.006
LA 18:2*n*-6	0.100 ± 0.009	0.76 ± 0.01	1.07 ± 0.04	0.91 ± 0.03
ARA 20:4*n*-6	0.097 ± 0.002	0.236 ± 0.005	0.257 ± 0.009	0.312 ± 0.008
Sum *n*-6	0.22 ± 0.01	1.06 ± 0.02	1.42 ± 0.05	1.23 ± 0.04
ALA 18:3*n*-3	0.020 ± 0.001	0.0240 ± 0.0001	0.067 ± 0.003	0.037 ± 0.006
EPA 20:5*n*-3	0.690 ± 0.007	0.02763 ± 0.0004	0.043 ± 0.003	0.74 ± 0.06
DHA 22:6*n*-3	1.78 ± 0.01	0.015 ± 0.001	0.037 ± 0.003	2.0 ± 0.1
Sum EPA+DHA	2.47 ± 0.02	0.043 ± 0.001	0.080 ± 0.005	2.7 ± 0.2
Sum *n*-3	2.61 ± 0.03	0.130 ± 0.002	0.22 ± 0.01	2.8 ± 0.2
Sum identified FAs	4.8 ± 0.1	2.44 ± 0.04	4.5 ± 0.2	7.2 ± 0.3
*n*-6:*n*-3 ratio	0.085 ± 0.005	8.2 ± 0.2	6.4 ± 0.1	0.43 ± 0.03
EPA:DHA ratio	0.388 ± 0.001	1.80 ± 0.05	2.2 ± 0.3	0.376 ± 0.008
ARA:EPA ratio	0.14 ± 0.001	8.53 ± 0.04	6.0 ± 0.3	0.43 ± 0.04
Neutral lipid fraction				
Sum SFA	76 ± 1	76 ± 2	71 ± 2	72 ± 0.5
Sum MUFA	58 ± 2	60 ± 2	63 ± 2	57.7 ± 0.8
LA 18:2*n*-6	24 ± 1	23.1 ± 0.4	22.1 ± 0.7	22.5 ± 0.5
ARA 20:4*n*-6	0.188 ± 0.007	0.302 ± 0.009	0.36 ± 0.02	0.32 ± 0.02
Sum *n*-6	24 ± 1	23.7 ± 0.4	23.0 ± 0.8	23.0 ± 0.5
ALA 18:3*n*-3	4.0 ± 0.2	3.8 ± 0.2	3.7 ± 0.1	3.86 ± 0.04
EPA 20:5*n*-3	0.15 ± 0.01	<0.01	1.17 ± 0.06	0.15 ± 0.01
DHA 22:6*n*-3	0.16 ± 0.01	<0.01	1.64 ± 0.09	0.096 ± 0.004
Sum EPA+DHA	0.31 ± 0.01	<0.01	2.8 ± 0.2	0.24 ± 0.01
Sum *n*-3	4.7 ± 0.2	4.1 ± 0.2	7.3 ± 0.3	4.51 ± 0.05
Sum identified FAs	164 ± 5	163 ± 4	164 ± 4	157 ± 2
*n*-6:*n*-3 ratio	5.2 ± 0.1	5.8 ± 0.3	3.2 ± 0.2	5.00 ± 0.06
EPA:DHA ratio	0.93 ± 0.15	*	0.725 ± 0.007	1.6 ± 0.1
ARA:EPA ratio	1.3 ± 0.1	*	0.31 ± 0.03	2.1 ± 0.1

Results are presented as means ± SEMs of three samples and indicate mg FAs in the polar and neutral lipid fractions/g Western diet. * not possible to calculate; EPA and DHA levels are under the limit of quantification (<0.01 mg/g). Abbreviations: SFA, saturated fatty acids; MUFA, monounsaturated fatty acids; LA, linoleic acid; ARA, arachidonic acid; ALA, alpha-linolenic acid; EPA, eicosapentaenoic acid; DHA, docosahexaenoic acid; FAs, fatty acids.

**Table 3:** Fatty acid composition in red blood cells.

Fatty Acid (mg/g)	Fresh Cod	Pork	Pork *n*-3 TAG	Pork *n*-3 PL
Sum SFA	1.56 ± 0.06	1.51 ± 0.04	1.47 ± 0.05	1.48 ± 0.04
Sum MUFA	0.69 ± 0.03	0.70 ± 0.02	0.67 ± 0.01	0.65 ± 0.02
LA 18:2*n*-6	0.48 ± 0.03	0.46 ± 0.02	0.44 ± 0.03	0.43 ± 0.02
ARA 20:4*n*-6	0.41 ± 0.01 ^a^	0.66 ± 0.01 ^b^	0.45 ± 0.01 ^ac^	0.464 ± 0.009 ^c^
Sum *n*-6	0.99 ± 0.05 ^a^	1.27 ± 0.03 ^b^	0.97 ± 0.03 ^a^	0.99 ± 0.02 ^a^
ALA 18:3*n*-3	<0.01	<0.01	<0.01	<0.01
EPA 20:5*n*-3	0.119 ± 0.005 ^a^	0.031 ± 0.001 ^b^	0.127 ± 0.004 ^a^	0.095 ± 0.002 ^c^
DHA 22:6*n*-3	0.39 ± 0.02 ^a^	0.224 ± 0.007 ^b^	0.354 ± 0.009 ^c^	0.381 ± 0.007 ^ac^
Sum EPA+DHA	0.51 ± 0.02 ^a^	0.255 ± 0.008 ^b^	0.48 ± 0.01 ^a^	0.477 ± 0.008 ^a^
Sum *n*-3	0.57 ± 0.02 ^a^	0.313 ± 0.008 ^b^	0.55 ± 0.01 ^a^	0.535 ± 0.009 ^a^
Sum identified FAs	3.8 ± 0.1	3.80 ± 0.09	3.7 ± 0.1	3.66 ± 0.08
*n*-6:*n*-3 ratio	1.73 ± 0.03 ^a^	4.08 ± 0.09 ^b^	1.76 ± 0.03 ^a^	1.85 ± 0.04 ^a^
ARA:EPA ratio	3.49 ± 0.09 ^a^	21.7 ± 0.8 ^b^	3.6 ± 0.1 ^a^	4.9 ± 0.2 ^c^

Results are presented as means ± SEMs (*n* = 10) and indicate mg FAs/g RBCs. Data were analyzed using one-way ANOVA followed by Fisher’s LSD post hoc tests. Different letters denote statistical significance (*p* ≤ 0.05) between the groups. Abbreviations: SFA, saturated fatty acids; MUFA, monounsaturated fatty acids; LA, linoleic acid; ARA, arachidonic acid; ALA, alpha-linolenic acid; EPA, eicosapentaenoic acid; DHA, docosahexaenoic acid; FAs, fatty acids; RBCs, red blood cells.

## Supplementary Materials

The following are available online at http://www.mdpi.com/2072-6643/10/6/695/s1; Figure S1: Effects of Western diets with different protein sources on glucose tolerance and glucose-stimulated insulin secretion in male C57BL/6J after 11 weeks on the experimental diets. As reference, a low fat fed group (*n* = 5) was included and is shown as a dotted line. (a) An oral glucose tolerance test (OGTT) was performed on mice fasted for 6 h. Blood glucose levels were recorded before (0) and at 15, 30, 60 and 120 min after oral administration of glucose (3 mg glucose/g lean mass). (b) Blood glucose area under the curve (AUC) and (c) incremental blood glucose area under the curve (iAUC) were calculated. (d) Blood was collected and plasma prepared for insulin measured before (0) and at 15, 30 and 120 min after oral administration of glucose. (e) Measurements of 6 h fasted blood glucose (f) 15 min, (g) 30 min, (h) and 120 min after the oral administration of glucose. (i) 6 h fasted plasma insulin (j) and plasma insulin levels at 15 min, (k) 30 min, (l) and 120 min after the oral administration of glucose. Data are presented as mean ± SEM (*n* = 10) and were analyses using one-way ANOVA followed by Fisher’s LSD post hoc test. Different letters denote statistical significance (*p* ≤ 0.05) between the groups; Figure S2: Effects of Western diets on fasting blood glucose levels after 10 weeks on experimental diets. Male C57BL/6J mice were fed Western diets containing either, fresh cod (fresh cod) or fresh pork (pork) as protein sources for 12 weeks, two pork diets was added n-3 PUFAs to the level of the fresh cod containing diet either as TAG-bound (pork n-3 TAG) or PL-bound (pork n-3 PL) bound n-3 EPA+DHA. As reference, a low fat fed group (*n* = 5) was included and is shown as a dotted line. Data are presented as mean ± SEM (*n* = 14–15) and were analyses using one-way ANOVA followed by Fisher’s LSD post hoc test. Different letters denote statistical significance (*p* ≤ 0.05) between the groups; Table S1: Lipid class composition in raw or heated fresh cod fillets; Table S2: Fatty acid composition in the polar and neutral lipid fractions isolated from raw or heated fresh cod fillets; Table S3: Fatty acid composition in the polar and neutral lipid fractions isolated from freeze dried fresh and frozen cod fillets; Table S4: Compositions of the diets in experiment 1; Table S5: Fatty acid composition in the polar and neutral lipid fractions isolated from Western diets; Table S6: Lipid class composition in freeze dried fresh cod fillets; Table S7: Fatty acid compositions in the polar and neutral lipid fractions isolated from freeze dried fresh cod fillets; Table S8: Fatty acid compositions in the polar and neutral lipid fractions isolated from soybean oil, PL-soybean oil and cod liver oil; Table S9: Compositions of the diets in experiment 2; Table S10: Fatty acid composition in the polar and neutral lipid fractions isolated from mouse liver; Table S11: Fatty acid composition in red blood cells.

## References

[1] Mozaffarian D. (2016). Dietary and policy priorities for cardiovascular disease, diabetes, and obesity: A comprehensive review. Circulation.

[2] Smith J.D., Hou T., Ludwig D.S., Rimm E.B., Willett W., Hu F.B., Mozaffarian D. (2015). Changes in intake of protein foods, carbohydrate amount and quality, and long-term weight change: Results from 3 prospective cohorts. Am. J. Clin. Nutr..

[3] Holm J.B., Ronnevik A., Tastesen H.S., Fjaere E., Fauske K.R., Liisberg U., Madsen L., Kristiansen K., Liaset B. (2016). Diet-induced obesity, energy metabolism and gut microbiota in c57bl/6j mice fed western diets based on lean seafood or lean meat mixtures. J. Nutr. Biochem..

[4] Liisberg U., Fauske K.R., Kuda O., Fjaere E., Myrmel L.S., Norberg N., Froyland L., Graff I.E., Liaset B., Kristiansen K. (2016). Intake of a western diet containing cod instead of pork alters fatty acid composition in tissue phospholipids and attenuates obesity and hepatic lipid accumulation in mice. J. Nutr. Biochem..

[5] Lavigne C., Marette A., Jacques H. (2000). Cod and soy proteins compared with casein improve glucose tolerance and insulin sensitivity in rats. Am. J. Physiol. Endocrinol. Metab..

[6] Lavigne C., Tremblay F., Asselin G., Jacques H., Marette A. (2001). Prevention of skeletal muscle insulin resistance by dietary cod protein in high fat-fed rats. Am. J. Physiol. Endocrinol. Metab..

[7] Tastesen H.S., Keenan A.H., Madsen L., Kristiansen K., Liaset B. (2014). Scallop protein with endogenous high taurine and glycine content prevents high-fat, high-sucrose-induced obesity and improves plasma lipid profile in male c57bl/6j mice. Amino. Acids.

[8] Liisberg U., Myrmel L.S., Fjaere E., Ronnevik A.K., Bjelland S., Fauske K.R., Holm J.B., Basse A.L., Hansen J.B., Liaset B. (2016). The protein source determines the potential of high protein diets to attenuate obesity development in c57bl/6j mice. Adipocyte.

[9] Lie Ø., Lambertsen G. (1991). Fatty acid composition of glycerophospholipids in seven tissues of cod (*Gadus morhua*), determined by combined high-performance liquid chromatography and gas chromatography. J. Chromatogr. B Biomed. Sci. Appl..

[10] Murru E., Banni S., Carta G. (2013). Nutritional properties of dietary omega-3-enriched phospholipids. Biomed. Res. Int..

[11] Rossmeisl M., Jilkova Z.M., Kuda O., Jelenik T., Medrikova D., Stankova B., Kristinsson B., Haraldsson G.G., Svensen H., Stoknes I. (2012). Metabolic effects of n-3 pufa as phospholipids are superior to triglycerides in mice fed a high-fat diet: Possible role of endocannabinoids. PLoS ONE.

[12] Roiha I.S., Jonsson A., Backi C.J., Lunestad B.T., Karlsdottir M.G. (2018). A comparative study of quality and safety of atlantic cod (*Gadus morhua*) fillets during cold storage, as affected by different thawing methods of pre-rigor frozen headed and gutted fish. J. Sci. Food Agric..

[13] Bligh E., Scott M.A. (1966). Lipids of cod muscle and the effect of frozen storage. J. Fish. Board Can..

[14] Olley J., Lovern J. (1960). Phospholipid hydrolysis in cod flesh stored at various temperatures. J. Sci. Food Agric..

[15] Anderson M.L., Ravesi E.M. (1969). Reaction of free fatty acids with protein in cod muscle frozen and stored at−29 c after aging in ice. J. Fish. Board Can..

[16] Dyer W., Fraser D.I. (1959). Proteins in fish muscle. 13. Lipid hydrolysis. J. Fish. Board Can..

[17] Badii F., Howell N.K. (2002). Changes in the texture and structure of cod and haddock fillets during frozen storage. Food Hydrocoll..

[18] Badii F., Howell N.K. (2002). A comparison of biochemical changes in cod (*Gadus morhua*) and haddock (*Melanogrammus aeglefinus*) fillets during frozen storage. J. Sci. Food Agric..

[19] Jordal A.E., Lie Ø., Torstensen B. (2007). Complete replacement of dietary fish oil with a vegetable oil blend affect liver lipid and plasma lipoprotein levels in atlantic salmon (*Salmo salar* L.). Aquacult. Nutr..

[20] Torstensen B.E., Frøyland L., Ørnsrud R., Lie Ø. (2004). Tailoring of a cardioprotective muscle fatty acid composition of atlantic salmon (*Salmo salar*) fed vegetable oils. Food Chem..

[21] Mariotti F., Tome D., Mirand P.P. (2008). Converting nitrogen into protein—Beyond 6.25 and jones’ factors. Crit. Rev. Food Sci. Nutr..

[22] Halldorsdottir S., Carmody J., Boozer C.N., Leduc C.A., Leibel R.L. (2009). Reproducibility and accuracy of body composition assessments in mice by dual energy x-ray absorptiometry and time domain nuclear magnetic resonance. Int. J. Body Compos. Res..

[23] Andrikopoulos S., Blair A.R., Deluca N., Fam B.C., Proietto J. (2008). Evaluating the glucose tolerance test in mice. Am. J. Physiol. Endocrinol. Metab..

[24] Batetta B., Griinari M., Carta G., Murru E., Ligresti A., Cordeddu L., Giordano E., Sanna F., Bisogno T., Uda S. (2009). Endocannabinoids may mediate the ability of (n-3) fatty acids to reduce ectopic fat and inflammatory mediators in obese zucker rats. J. Nutr..

[25] Vigerust N.F., Bjorndal B., Bohov P., Brattelid T., Svardal A., Berge R.K. (2013). Krill oil versus fish oil in modulation of inflammation and lipid metabolism in mice transgenic for tnf-alpha. Eur. J. Nutr..

[26] Tillander V., Bjorndal B., Burri L., Bohov P., Skorve J., Berge R.K., Alexson S.E. (2014). Fish oil and krill oil supplementations differentially regulate lipid catabolic and synthetic pathways in mice. Nutr. Metab..

[27] Burri L., Berge K., Wibrand K., Berge R.K., Barger J.L. (2011). Differential effects of krill oil and fish oil on the hepatic transcriptome in mice. Front. Genet..

[28] Awada M., Meynier A., Soulage C.O., Hadji L., Geloen A., Viau M., Ribourg L., Benoit B., Debard C., Guichardant M. (2013). N-3 pufa added to high-fat diets affect differently adiposity and inflammation when carried by phospholipids or triacylglycerols in mice. Nutr. Metab..

[29] Tou J.C., Jaczynski J., Chen Y.C. (2007). Krill for human consumption: Nutritional value and potential health benefits. Nutr. Rev..

[30] Vance D.E. (2014). Phospholipid methylation in mammals: From biochemistry to physiological function. Biochim. Biophys. Acta..

[31] Niki E. (2014). Role of vitamin e as a lipid-soluble peroxyl radical scavenger: In vitro and in vivo evidence. Free Radic. Biol. Med..

[32] Ni Y., Nagashimada M., Zhuge F., Zhan L., Nagata N., Tsutsui A., Nakanuma Y., Kaneko S., Ota T. (2015). Astaxanthin prevents and reverses diet-induced insulin resistance and steatohepatitis in mice: A comparison with vitamin e. Sci. Rep..

[33] Tandy S., Chung R.W., Wat E., Kamili A., Berge K., Griinari M., Cohn J.S. (2009). Dietary krill oil supplementation reduces hepatic steatosis, glycemia, and hypercholesterolemia in high-fat-fed mice. J. Agric. Food Chem..

[34] Liu X., Cui J., Li Z., Xu J., Wang J., Xue C., Wang Y. (2014). Comparative study of dha-enriched phospholipids and epa-enriched phospholipids on metabolic disorders in diet-induced-obese c57bl/6j mice. Eur. J. Lipid Sci. Technol..

[35] Madsen L., Frøyland L., Dyrøy E., Helland K., Berge R.K. (1998). Docosahexaenoic and eicosapentaenoic acids are differently metabolized in rat liver during mitochondria and peroxisome proliferation. J. Lipid Res..

[36] Lopez-Vicario C., Rius B., Alcaraz-Quiles J., Garcia-Alonso V., Lopategi A., Titos E., Claria J. (2016). Pro-resolving mediators produced from epa and dha: Overview of the pathways involved and their mechanisms in metabolic syndrome and related liver diseases. Eur. J. Pharmacol..

[37] Frøyland L., Madsen L., Vaagenes H., Totland G., Auwerx J., Kryvi H., Staels B., Berge R. (1997). Mitochondrion is the principal target for nutritional and pharmacological control of triglyceride metabolism. J. Lipid Res..

[38] Alvheim A.R., Torstensen B.E., Lin Y.H., Lillefosse H.H., Lock E.-J., Madsen L., Hibbeln J.R., Malde M.K. (2013). Dietary linoleic acid elevates endogenous 2-arachidonoylglycerol and anandamide in atlantic salmon (*Salmo salar* L.) and mice, and induces weight gain and inflammation in mice. Br. J. Nutr..

[39] Midtbø L.K., Borkowska A.G., Bernhard A., Rønnevik A.K., Lock E.-J., Fitzgerald M.L., Torstensen B.E., Liaset B., Brattelid T., Pedersen T.L. (2015). Intake of farmed atlantic salmon fed soybean oil increases hepatic levels of arachidonic acid-derived oxylipins and ceramides in mice. J. Nutr. Biochem..

[40] Midtbø L.K., Ibrahim M.M., Myrmel L.S., Aune U.L., Alvheim A.R., Liland N.S., Torstensen B.E., Rosenlund G., Liaset B., Brattelid T. (2013). Intake of farmed atlantic salmon fed soybean oil increases insulin resistance and hepatic lipid accumulation in mice. PLoS ONE.

[41] Salem N., Kuratko C.N. (2014). A reexamination of krill oil bioavailability studies. Lipids Health Dis..

[42] Fischer A.W., Cannon B., Nedergaard J. (2018). Optimal housing temperatures for mice to mimic the thermal environment of humans: An experimental study. Mol. Metab..

[43] Sadurskis A., Dicker A., Cannon B., Nedergaard J. (1995). Polyunsaturated fatty acids recruit brown adipose tissue: Increased ucp content and nst capacity. Am. J. Physiol..

[44] Kim M., Goto T., Yu R., Uchida K., Tominaga M., Kano Y., Takahashi N., Kawada T. (2015). Fish oil intake induces ucp1 upregulation in brown and white adipose tissue via the sympathetic nervous system. Sci. Rep..

[45] Cannon B., Nedergaard J. (2004). Brown adipose tissue: Function and physiological significance. Physiol. Rev..

[46] Feldmann H.M., Golozoubova V., Cannon B., Nedergaard J. (2009). Ucp1 ablation induces obesity and abolishes diet-induced thermogenesis in mice exempt from thermal stress by living at thermoneutrality. Cell Metab..

[47] Goldgof M., Xiao C., Chanturiya T., Jou W., Gavrilova O., Reitman M.L. (2014). The chemical uncoupler 2,4-dinitrophenol (dnp) protects against diet-induced obesity and improves energy homeostasis in mice at thermoneutrality. J. Biol. Chem..

[48] Ziętak M., Kovatcheva-Datchary P., Markiewicz L.H., Ståhlman M., Kozak L.P., Bäckhed F. (2016). Altered microbiota contributes to reduced diet-induced obesity upon cold exposure. Cell Metab..

[49] Rossmeisl M., Medrikova D., Van Schothorst E.M., Pavlisova J., Kuda O., Hensler M., Bardova K., Flachs P., Stankova B., Vecka M. (2014). Omega-3 phospholipids from fish suppress hepatic steatosis by integrated inhibition of biosynthetic pathways in dietary obese mice. Biochim. Biophys. Acta BBA Mol. Cell Biol. Lipids.

[50] Dyer W. (1951). Protein denaturation in frozen and stored fish. J. Food Sci..

[51] Shenouda S.Y. (1980). Theories of protein denaturation during frozen storage of fish flesh. Adv. Food Res..

