# Supplementary material for: Effects of Frozen Storage on Phospholipid Content in Atlantic Cod Fillets and the Influence on Diet-Induced Obesity in Mice
Source: Nutrients. 2018 May 30;10(6):695. doi: 10.3390/nu10060695 (PMC6024676; doi:10.3390/nu10060695)
Supplement: Supplementary file 1 [file nutrients-10-00695-s001.zip › Figure S1. Effects of Western diets with different protein sources on glucose tolerance and glucose-stimulated insulin secretion.docx]

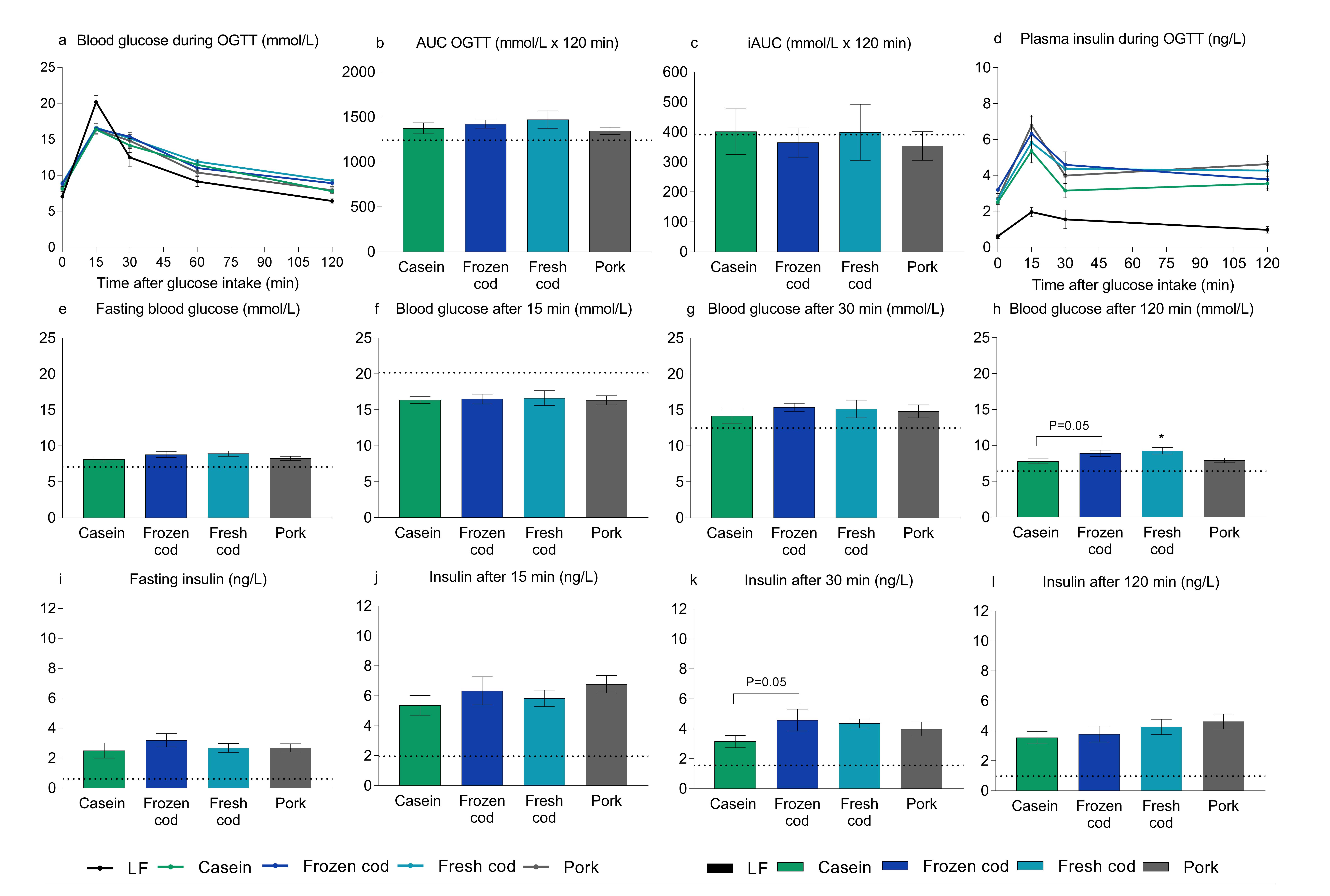


**Figure S1.** Effects of Western diets with different protein sources on glucose tolerance and glucose-stimulated insulin secretion in male C57BL/6J after 11 weeks on the experimental diets. As reference, a low fat fed group (n=5) was included and is shown as a dotted line. (**a**) An oral glucose tolerance test (OGTT) was performed on mice fasted for 6 h. Blood glucose levels were recorded before (0) and at 15, 30, 60 and 120 minutes after oral administration of glucose (3 mg glucose/g lean mass). (**b**) Blood glucose area under the curve (AUC) and (**c**) incremental blood glucose area under the curve (iAUC) were calculated. (**d**) Blood was collected and plasma prepared for insulin measured before (0) and at 15, 30 and 120 minutes after oral administration of glucose. (**e**) Measurements of 6 h fasted blood glucose (**f**) 15 min, (**g**) 30 min, (h) and 120 min after the oral administration of glucose. (**i**) 6 h fasted plasma insulin (**j**) and plasma insulin levels at 15 min, (**k**) 30 min, (**l**) and 120 min after the oral administration of glucose. Data are presented as mean ± SEM (n=10) and were analyses using one-way ANOVA followed by Fisher’s LSD post hoc test. Different letters denote statistical significance (P=<0.05) between the groups.
