# Supplementary material for: Effects of Frozen Storage on Phospholipid Content in Atlantic Cod Fillets and the Influence on Diet-Induced Obesity in Mice
Source: Nutrients. 2018 May 30;10(6):695. doi: 10.3390/nu10060695 (PMC6024676; doi:10.3390/nu10060695)
Supplement: Supplementary file 1 [file nutrients-10-00695-s001.zip › Figure S2. Effects of Western diets on fasting blood glucose levels.docx]

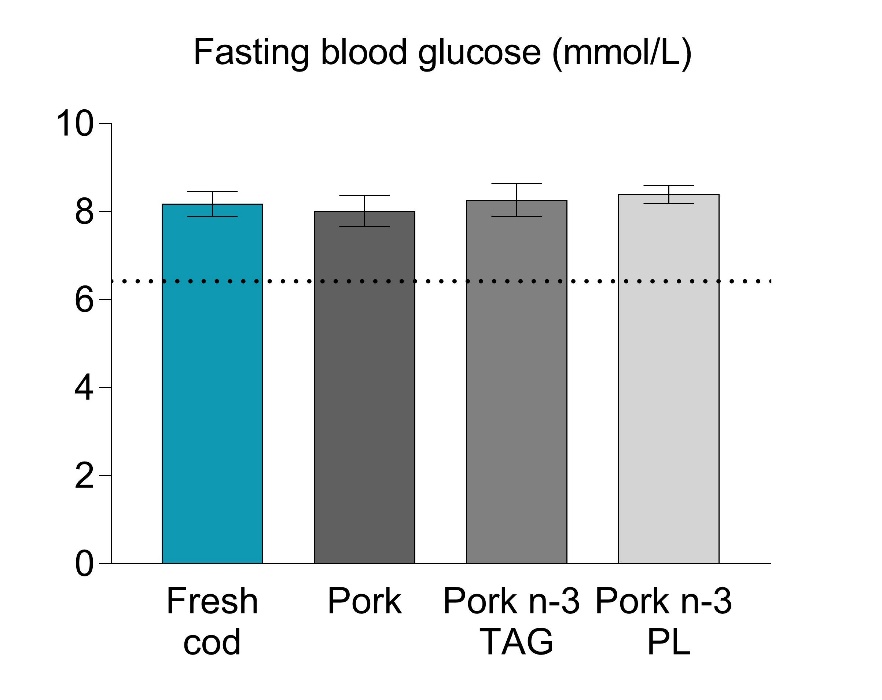


**Figure S2.** Effects of Western diets on fasting blood glucose levels after 10 weeks on experimental diets. Male C57BL/6J mice were fed Western diets containing either, fresh cod (fresh cod) or fresh pork (pork) as protein sources for 12 weeks, two pork diets was added n-3 PUFAs to the level of the fresh cod containing diet either as TAG-bound (pork n-3 TAG) or PL-bound (pork n-3 PL) bound n-3 EPA+DHA. As reference, a low fat fed group (n=5) was included and is shown as a dotted line. Data are presented as mean ± SEM (n=14-15) and were analyses using one-way ANOVA followed by Fisher’s LSD post hoc test. Different letters denote statistical significance (P=<0.05) between the groups.
