# Supplementary material for: Effects of Frozen Storage on Phospholipid Content in Atlantic Cod Fillets and the Influence on Diet-Induced Obesity in Mice
Source: Nutrients. 2018 May 30;10(6):695. doi: 10.3390/nu10060695 (PMC6024676; doi:10.3390/nu10060695)
Supplement: Supplementary file 1 [file nutrients-10-00695-s001.zip › Table S1. Lipid class composition in raw or heated fresh cod fillets.docx]

**Table S1.** Lipid class composition in raw or heated fresh cod fillets

|  | **Raw fresh cod fillets** | |  | **Heated fresh cod fillets** | |
| --- | --- | --- | --- | --- | --- |
| **Lipid class** | **mg/g** | **%** |  | **mg/g** | **%** |
| PC | 2.90 ± 0.04 | 51 |  | 5.25 ± 0.09 | 50.1 |
| PE | 1.06 ± 0.06 | 18.7 |  | 2.05 ± 0.03 | 19.5 |
| PI | 0.21 ± 0.01 | 3.6 |  | 0.461 ± 0.007 | 4.39 |
| PS | 0.07 ± 0.03 | 1.2 |  | 0.362 ± 0.009 | 3.45 |
| LPC | 0.28 ± 0.01 | 5.0 |  | 0.27 ± 0.02 | 2.5 |
| SM | 0.171 ± 0.007 | 3.0 |  | 0.335 ± 0.008 | 3.19 |
| CL | 0.033 ± 0.003 | 0.576 |  | 0.080 ± 0.007 | 0.76 |
| Sum polar lipids | 4.7 ± 0.1 | 83 |  | 8.80 ± 0.03 | 83.9 |
| FFA | 0.72 ± 0.03 | 12.7 |  | 0.66 ± 0.04 | 6.3 |
| CHOL | 0.48 ± 0.03 | 8.4 |  | 0.81 ± 0.03 | 7.8 |
| TAG | 0.02 ± 0.01 | 0.3 |  | 0.16 ± 0.02 | 1.5 |
| DAG | <0.01 | <0.01 |  | <0.01 | <0.01 |
| CE | <0.01 | <0.01 |  | <0.01 | <0.01 |
| Sum neutral lipids | 1.0 ± 0.3 | 17 |  | 1.69 ± 0.03 | 16.1 |
| Sum Lipids | 5.7 ± 0.4 |  |  | 10.49 ± 0.02 |  |
| Polar lipids:FFA ratio | 6.57 ± 0.02 |  |  | 13.3 ± 0.7 |  |

Results are presented as mean ± SEM of three samples and indicate mg lipids/g and percent lipid class of total lipids in the raw or heated fresh cod fillets Abbreviations: PC; Phosphatidylcholine, PE; Phosphatidylethanolamine, PI; Phosphatidylinositol, PS; Phosphatidylserin, LPC; Lysophosphatidylcholine, SM; Sphingomyelin , CL; Cardiolipin, FFA; Free fatty acid, CHOL; Cholesterol, TAG; Triacylglycerol, DAG; Diacylglycerol, CE; Cholesteryl ester.
