# Supplementary material for: Effects of Frozen Storage on Phospholipid Content in Atlantic Cod Fillets and the Influence on Diet-Induced Obesity in Mice
Source: Nutrients. 2018 May 30;10(6):695. doi: 10.3390/nu10060695 (PMC6024676; doi:10.3390/nu10060695)
Supplement: Supplementary file 1 [file nutrients-10-00695-s001.zip › Table S10. Fatty acid composition in the polar and neutral lipid fractions isolated from mouse liver.docx]

**Table S10.** Fatty acid composition in the polar and neutral lipid fractions isolated from mouse liver

| **Fatty acid (mg/g)** | **Frozen cod** | **Fresh cod** | **Pork** |
| --- | --- | --- | --- |
| Polar lipid fraction |  |  |  |
| Sum SFA | 6.2 ± 0.2 ^ab^ | 6.5 ± 0.2 ^a^ | 5.7 ± 0.3 ^b^ |
| Sum MUFA | 2.73 ± 0.06 | 2.8 ± 0.1 | 2.7 ± 0.1 |
| LA 18:2n-6 | 2.6 ± 0.1 | 2.7 ± 0.1 | 2.4 ± 0.2 |
| ARA 20:4n-6 | 1.52 ± 0.04 ^a^ | 1.63 ± 0.05 ^a^ | 2.8 ± 0.1 ^b^ |
| Sum n-6 | 4.6 ± 0.2 ^a^ | 4.9 ± 0.2 ^a^ | 5.8 ± 0.3 ^b^ |
| ALA 18:3n-3 | 0.033 ± 0.002 ^a^ | 0.033 ± 0.003 ^a^ | 0.023 ± 0.002 ^b^ |
| EPA 20:5n-3 | 0.79 ± 0.04 ^a^ | 0.83 ± 0.05 ^a^ | 0.18 ± 0.01 ^b^ |
| DHA 22:6n-3 | 2.7 ± 0.1 ^a^ | 2.77 ± 0.09 ^a^ | 1.62 ± 0.07 ^b^ |
| Sum EPA+DHA | 3.5 ± 0.1 ^a^ | 3.6 ± 0.1 ^a^ | 1.80 ± 0.08 ^b^ |
| Sum n-3 | 3.7 ± 0.1 ^a^ | 3.8 ± 0.1 ^a^ | 1.93 ± 0.09 ^b^ |
| Sum identified FAs | 17.2 ± 0.6 | 18.0 ± 0.7 | 16.2 ± 0.7 |
| n-6:n-3 ratio | 1.24 ± 0.02 ^a^ | 1.29 ± 0.02 ^a^ | 2.99 ± 0.06 ^b^ |
| ARA:EPA ratio | 1.95 ± 0.08 ^a^ | 2.01 ± 0.08 ^a^ | 15.6 ± 0.6 ^b^ |
|  |  |  |  |
| Neutral lipid fraction |  |  |  |
| Sum SFA | 24 ± 5 | 27 ± 5 | 33 ± 6 |
| Sum MUFA | 63 ± 15 | 67 ± 13 | 88 ± 16 |
| LA 18:2n-6 | 10 ± 2 | 12 ± 2 | 13 ± 1 |
| ARA 20:4n-6 | 0.29 ± 0.05 ^a^ | 0.34 ± 0.04 ^a^ | 0.66 ± 0.06 ^b^ |
| Sum n-6 | 11 ± 2 | 12 ± 2 | 14 ± 2 |
| ALA 18:3n-3 | 1.4 ± 0.3 | 1.5 ± 0.2 | 1.2 ± 0.1 |
| EPA 20:5n-3 | 0.9 ± 0.1 ^a^ | 1.1 ± 0.1 ^a^ | 0.28 ± 0.03 ^b^ |
| DHA 22:6n-3 | 4.7 ± 0.6 ^a^ | 5.4 ± 0.5 ^a^ | 1.2 ± 0.1 ^b^ |
| Sum EPA+DHA | 5.7 ± 0.7 ^a^ | 6.5 ± 0.6 ^a^ | 1.4 ± 0.1 ^b^ |
| Sum n-3 | 8 ± 1 ^a^ | 9.4 ± 0.9 ^a^ | 3.4 ± 0.3 ^b^ |
| Sum identified FAs | 108 ± 23 | 116 ± 21 | 139 ± 23 |
| n-6:n-3 ratio | 1.28 ± 0.09 ^a^ | 1.30 ± 0.06 ^a^ | 4.3 ± 0.2 ^b^ |
| ARA:EPA ratio | 0.32 ± 0.02 ^a^ | 0.30 ± 0.02 ^a^ | 2.41 ± 0.09 ^b^ |

Results are presented as mean ± SEM (n=10) and indicate mg FAs in the polar and neutral lipid fractions/g liver. Results are analyzed using one-way ANOVA followed by Fisher’s LSD post hoc test. Significant differences (P<0.05) between the groups are marked with different letters. Abbreviations: SFA; saturated fatty acids, MUFA; monounsaturated fatty acids, LA; linoleic acid, ARA; arachidonic acid, ALA; alpha-linolenic acid, EPA; eicosapentaenoic acid, DHA; docosahexaenoic acid, FAs; fatty acids.
