# Supplementary material for: Effects of Frozen Storage on Phospholipid Content in Atlantic Cod Fillets and the Influence on Diet-Induced Obesity in Mice
Source: Nutrients. 2018 May 30;10(6):695. doi: 10.3390/nu10060695 (PMC6024676; doi:10.3390/nu10060695)
Supplement: Supplementary file 1 [file nutrients-10-00695-s001.zip › Table S11. Fatty acid composition in red blood cells.docx]

**Table S11**. Fatty acid composition in red blood cells

| **Fatty acid (mg/g)** | **Frozen cod** | **Fresh cod** | **Pork** |
| --- | --- | --- | --- |
| Sum SFA | 1.27 ± 0.04 | 1.23 ± 0.07 | 1.26 ± 0.05 |
| Sum MUFA | 0.54 ± 0.02 | 0.53 ± 0.03 | 0.55 ± 0.02 |
| LA 18:2n-6 | 0.35 ± 0.01 | 0.33 ± 0.02 | 0.32 ± 0.02 |
| ARA 20:4n-6 | 0.264 ± 0.006 ^a^ | 0.24 ± 0.01 ^a^ | 0.47 ± 0.01 ^b^ |
| Sum n-6 | 0.66 ± 0.01 ^a^ | 0.61 ± 0.04 ^a^ | 0.87 ± 0.02 ^b^ |
| ALA 18:3n-3 | <0.01 | <0.01 | <0.01 |
| EPA 20:5n-3 | 0.104 ± 0.004 ^a^ | 0.097 ± 0.006 ^a^ | 0.022 ± 0.001 ^b^ |
| DHA 22:6n-3 | 0.264 ± 0.007 ^a^ | 0.24 ± 0.02 ^a^ | 0.164 ± 0.009 ^b^ |
| Sum EPA+DHA | 0.37 ± 0.01 ^a^ | 0.34 ± 0.02 ^a^ | 0.19 ± 0.01 ^b^ |
| Sum n-3 | 0.40 ± 0.01 ^a^ | 0.37 ± 0.02 ^a^ | 0.22 ± 0.01 ^b^ |
| Sum identified FAs | 2.87 ± 0.07 | 2.7 ± 0.2 | 2.91 ± 0.09 |
| n-6:n-3 ratio | 1.67 ± 0.03 ^a^ | 1.68 ± 0.06 ^a^ | 4.0 ± 0.2 ^b^ |
| ARA:EPA ratio | 2.6 ± 0.1 ^a^ | 2.43 ± 0.07 ^a^ | 21.9 ± 0.8 ^b^ |

Results are presented as mean ± SEM and indicate mg FA/g red blood cells. Data were analyzed using one-way ANOVA followed by Fisher’s LSD post hoc test. Different letters denote statistical significance (P=<0.05) between the groups. Abbreviations: SFA; saturated fatty acids, MUFA; monounsaturated fatty acids, LA; linoleic acid, ARA; arachidonic acid, ALA; alpha-linolenic acid, EPA; eicosapentaenoic acid, DHA; docosahexaenoic acid, FAs; fatty acids.
