# Supplementary material for: Effects of Frozen Storage on Phospholipid Content in Atlantic Cod Fillets and the Influence on Diet-Induced Obesity in Mice
Source: Nutrients. 2018 May 30;10(6):695. doi: 10.3390/nu10060695 (PMC6024676; doi:10.3390/nu10060695)
Supplement: Supplementary file 1 [file nutrients-10-00695-s001.zip › Table S2. Fatty acid composition in polar and neutral lipid fractions isolated from raw or heated fresh cod fillets.docx]

**Table S2.** Fatty acid composition in the polar and neutral lipid fractions isolated from raw or heated fresh cod fillets

|  | **Raw fresh cod fillets** | |  | **Heated fresh cod fillets** | |
| --- | --- | --- | --- | --- | --- |
| **Fatty acid** | **mg/g** | **%** |  | **mg/g** | **%** |
| Polar lipid fraction |  |  |  |  |  |
| Sum SFA | 0.63 ± 0.08 | 24.41 |  | 1.05 ± 0.08 | 24.77 |
| Sum MUFA | 0.37 ± 0.05 | 14.2 |  | 0.54 ± 0.04 | 12.785 |
| LA 18:2n-6 | 0.024 ± 0.003 | 0.95 |  | 0.036 ± 0.002 | 0.86 |
| ARA 20:4n-6 | 0.050 ± 0.006 | 1.93 |  | 0.085 ± 0.006 | 2.01 |
| Sum n-6 | 0.09 ± 0.01 | 3.55 |  | 0.15 ± 0.01 | 3.49 |
| ALA 18:3n-3 | <0.01 | <0.01 |  | <0.01 | <0.01 |
| EPA 20:5n-3 | 0.39 ± 0.05 | 15.4 |  | 0.61 ± 0.05 | 14.37 |
| DHA 22:6n-3 | 1.0 ± 0.1 | 40.0 |  | 1.8 ± 0.1 | 42.31 |
| Sum EPA + DHA | 1.4 ± 0.2 | 55.4 |  | 2.4 ± 0.2 | 56.67 |
| Sum n-3 | 1.5 ± 0.2 | 57.8 |  | 2.5 ± 0.2 | 58.88 |
| Sum identified FAs | 2.6 ± 0.3 |  |  | 4.2 ± 0.3 |  |
| n-6:n-3 ratio | 0.062 ± 0.001 |  |  | 0.0592 ± 0.0004 |  |
| EPA:DHA | 0.385 ± 0.005 |  |  | 0.3396 |  |
| ARA:EPA ratio | 0.1255 ± 0.0004 |  |  | 0.1400 ± 0.0008 |  |
|  |  |  |  |  |  |
| Neutral lipid fraction |  |  |  |  |  |
| Sum SFA | 0.19 ± 0.03 | 24.5 |  | 0.18 ± 0.01 | 22.8 |
| Sum MUFA | 0.15 ± 0.02 | 19.7 |  | 0.18 ± 0.02 | 22.8 |
| LA 18:2n-6 | <0.01 | <0.01 |  | <0.01 | <0.01 |
| ARA 20:4n-6 | 0.012 ± 0.002 | 1.60 |  | 0.016 ± 0.001 | 1.988 |
| Sum n-6 | 0.024 ± 0.004 | 3.17 |  | 0.029 ± 0.002 | 3.65 |
| ALA 18:3n-3 | <0.01 | <0.01 |  | <0.01 | <0.01 |
| EPA 20:5n-3 | 0.15 ± 0.02 | 19.2 |  | 0.156 ± 0.010 | 19.5 |
| DHA 22:6n-3 | 0.23 ± 0.04 | 30.0 |  | 0.22 ± 0.02 | 27.47 |
| Sum EPA + DHA | 0.38 ± 0.06 | 49.2 |  | 0.38 ± 0.03 | 47.0 |
| Sum n-3 | 0.40 ± 0.07 | 52.5 |  | 0.40 ± 0.03 | 50.6 |
| Sum identified FAs | 0.8 ± 0.1 |  |  | 0.80 ± 0.06 |  |
| n-6:n-3 ratio | 0.060 ± 0.001 |  |  | 0.0722 ± 0.0009 |  |
| EPA:DHA | 0.64 ± 0.01 |  |  | 0.710 ± 0.005 |  |
| ARA:EPA ratio | 0.084 ± 0.002 |  |  | 0.1019 ± 0.0006 |  |

Results are presented as mean ± SEM of three samples and indicate mg FAs/g and percent FAs of total identified FAs in the raw or heated fresh cod fillets. Abbreviations: SFA; saturated fatty acids, MUFA; monounsaturated fatty acids, LA; linoleic acid, ARA; arachidonic acid, ALA; alpha-linolenic acid, EPA; eicosapentaenoic acid, DHA; docosahexaenoic acid, FAs; fatty acids.
