# Supplementary material for: Effects of Frozen Storage on Phospholipid Content in Atlantic Cod Fillets and the Influence on Diet-Induced Obesity in Mice
Source: Nutrients. 2018 May 30;10(6):695. doi: 10.3390/nu10060695 (PMC6024676; doi:10.3390/nu10060695)
Supplement: Supplementary file 1 [file nutrients-10-00695-s001.zip › Table S3. Fatty acid composition in polar and neutral lipid fractions isolated from freeze dried fresh or frozen cod fillet.docx]

**Table S3.** Fatty acid composition in the polar and neutral lipid fractions isolated from freeze dried fresh and frozen cod fillets

|  | **Freeze dried frozen cod** | |  | **Freeze dried fresh cod** | |
| --- | --- | --- | --- | --- | --- |
| **Fatty acid** | **mg/g** | **%** |  | **mg/g** | **%** |
| Polar lipid fraction |  |  |  |  |  |
| Sum SFA | 4.1 ± 0.7 | 25.1 |  | 4.8 ± 0.7 | 25.5 |
| Sum MUFA | 2.3 ± 0.3 | 13.9 |  | 2.6 ± 0.4 | 13.8 |
| LA 18:2n-6 | 0.14 ± 0.02 | 0.884 |  | 0.17 ± 0.03 | 0.89 |
| ARA 20:4n-6 | 0.29 ± 0.04 | 1.78 |  | 0.33 ± 0.04 | 1.75 |
| Sum n-6 | 0.52 ± 0.07 | 3.21 |  | 0.61 ± 0.06 | 3.3 |
| ALA 18:3n-3 | 0.022 ± 0.003 | 0.137 |  | 0.027 ± 0.005 | 0.143 |
| EPA 20:5n-3 | 2.2 ± 0.4 | 13.4 |  | 2.7 ± 0.4 | 14.0 |
| DHA 22:6n-3 | 7 ± 1 | 42.5 |  | 8 ± 1 | 41.4 |
| Sum EPA + DHA | 9 ± 1 | 55.87 |  | 10 ± 1 | 55.4 |
| Sum n-3 | 9 ± 2 | 57.79 |  | 11 ± 1 | 57.3 |
| Sum identified FAs | 16 ± 3 |  |  | 19 ± 3 |  |
| n-6:n-3 ratio | 0.055 ± 0.001 |  |  | 0.058 ± 0.002 |  |
| EPA:DHA ratio | 0.32 ± 0.02 |  |  | 0.34 ± 0.02 |  |
| ARA:EPA ratio | 0.133 ± 0.008 |  |  | 0.125 ± 0.007 |  |
|  |  |  |  |  |  |
| Neutral lipid fraction |  |  |  |  |  |
| Sum SFA | 2.5 ± 0.6 | 23.5 |  | 2.0 ± 0.5 | 23.1 |
| Sum MUFA | 1.8 ± 0.3 | 18 |  | 1.6 ± 0.2 | 20 |
| LA 18:2n-6 | 0.12 ± 0.02 | 1.15 |  | 0.10 ± 0.02 | 1.17 |
| ARA 20:4n-6 | 0.23 ± 0.05 | 2.20 |  | 0.19 ± 0.05 | 2.19 |
| Sum n-6 | 0.41 ± 0.09 | 3.83 |  | 0.34 ± 0.09 | 3.9 |
| ALA 18:3n-3 | 0.025 ± 0.004 | 0.24 |  | 0.024 ± 0.002 | 0.29 |
| EPA 20:5n-3 | 2.2 ± 0.4 | 21.3 |  | 1.8 ± 0.4 | 21.9 |
| DHA 22:6n-3 | 3.3 ± 0.8 | 31 |  | 2.6 ± 0.8 | 28.8 |
| Sum EPA + DHA | 6 ± 1 | 52 |  | 4 ± 1 | 51 |
| Sum n-3 | 6 ± 1 | 55 |  | 5 ± 1 | 53 |
| Sum identified FAs | 11 ± 2 |  |  | 9 ± 2 |  |
| n-6:n-3 ratio | 0.0701 ± 0.0009 |  |  | 0.073 ± 0.003 |  |
| EPA:DHA ratio | 0.71 ± 0.08 |  |  | 0.77 ± 0.08 |  |
| ARA:EPA ratio | 0.104 ± 0.007 |  |  | 0.101 ± 0.007 |  |

Results are presented as mean ± SEM of three samples and indicate mg FAs /g and percent FAs of sum identified FAs in the freeze dried frozen or fresh cod fillets. Abbreviations: SFA; saturated fatty acids, MUFA; monounsaturated fatty acids, LA; linoleic acid, ARA; arachidonic acid, ALA; alpha-linolenic acid, EPA; eicosapentaenoic acid, DHA; docosahexaenoic acid, FAs; fatty acids.
