# Supplementary material for: Effects of Frozen Storage on Phospholipid Content in Atlantic Cod Fillets and the Influence on Diet-Induced Obesity in Mice
Source: Nutrients. 2018 May 30;10(6):695. doi: 10.3390/nu10060695 (PMC6024676; doi:10.3390/nu10060695)
Supplement: Supplementary file 1 [file nutrients-10-00695-s001.zip › Table S4. Compositions of the diets in experiment 1.docx]

**Table S4.** Compositions of the diets in experiment 1

| **Components (g/kg diet)** | **Casein** | **Frozen cod** | **Fresh cod** | **Pork** | **Low fat** |
| --- | --- | --- | --- | --- | --- |
| Casein | 222.0 |  |  |  | 222.0 |
| Freeze dried frozen cod fillets |  | 235.9 |  |  |  |
| Freeze dried fresh cod fillets |  |  | 242.9 |  |  |
| Freeze dried pork sirloins |  |  |  | 241.5 |  |
| Corn starch | 296.0 | 292.6 | 290.4 | 294.9 |  |
| Dextrin from potato starch | 100.0 | 100.0 | 100.0 | 100.0 | 507.5 |
| Sucrose | 80.0 | 80.0 | 80.0 | 80.0 | 100.0 |
| Soybean oil | 12.0 | 12.0 | 12.0 | 12.0 |  |
| Corn oil | 8.0 | 8.0 | 8.0 | 8.0 | 70.0 |
| Milk fat | 60.0 | 56.5 | 54.9 | 53.9 |  |
| Lard | 60.0 | 56.5 | 54.9 | 53.9 |  |
| Margarine | 60.0 | 56.5 | 54.9 | 53.9 |  |
| Cholesterol | 1.5 | 1.5 | 1.5 | 1.5 |  |
| Lipids from freeze dried frozen cod fillets |  | 10.6 |  |  |  |
| Lipids from freeze dried fresh cod fillets |  |  | 15.3 |  |  |
| Lipids from freeze dried pork sirloins |  |  |  | 18.4 |  |
| Analyzed |  |  |  |  |  |
| Energy (KJ/g diet) | 20.52 ± 0.01 | 20.28 ± 0.28 | 20.2 ± 0.2 | 20.5 ± 0.1 | 18.51 ± 0.03 |

All diets were supplemented with 0.014 g/kg t-Butylhydroquinone, 35 g/kg AIN93G mineral mix, 10 g/kg AIN93VX NCR95 compliant vitamin mix, 3g/kg L-cystine, 2.5g/kg choline bitartrate and 50g/kg cellulose. The amount of casein, freeze dried frozen and fresh cod fillets and pork sirloins added is based on measurements of nitrogen in the protein powders. Crude protein concentration was calculated using the formula N*6.15 for casein and N*5.60 for cod fillets and pork. The calculated contribution of lipids present in the protein sources is based on measurements of total lipid content in the protein powders measured with HPTLC. Analyzed values represents mean ± SEM of three samples.
