# Supplementary material for: Effects of Frozen Storage on Phospholipid Content in Atlantic Cod Fillets and the Influence on Diet-Induced Obesity in Mice
Source: Nutrients. 2018 May 30;10(6):695. doi: 10.3390/nu10060695 (PMC6024676; doi:10.3390/nu10060695)
Supplement: Supplementary file 1 [file nutrients-10-00695-s001.zip › Table S5. Fatty acid composition in polar- and neutral lipid fractions isolated from Western diets.docx]

**Table S5**. Fatty acid composition in the polar and neutral lipid fractions isolated from Western diets

|  | **Frozen cod** | |  | **Fresh cod** | |  | **Pork** | |
| --- | --- | --- | --- | --- | --- | --- | --- | --- |
| **Fatty acid** | **mg/g** | **%** |  | **mg/g** | **%** |  | **mg/g** | **%** |
| Polar lipid fraction |  |  |  |  |  |  |  |  |
| Sum SFA | 1.65 ± 0.03 | 31.5 |  | 2.20 ± 0.07 | 29.4 |  | 1.22 ± 0.04 | 45.9 |
| Sum MUFA | 1.12 ± 0.03 | 21.4 |  | 1.39 ± 0.06 | 18.6 |  | 0.91 ± 0.02 | 34.2 |
| LA 18:2n-6 | 0.32 ± 0.02 | 6.0 |  | 0.36 ± 0.03 | 4.7 |  | 0.430 ± 0.008 | 16.2 |
| ARA 20:4n-6 | 0.065 ± 0.002 | 1.25 |  | 0.099 ± 0.001 | 1.35 |  | <0.01 | <0.01 |
| Sum n-6 | 0.41 ± 0.02 | 7.9 |  | 0.49 ± 0.03 | 6.5 |  | 0.448 ± 0.009 | 16.8 |
| ALA 18:3n-3 | 0.054 ± 0.004 | 1.04 |  | 0.064 ± 0.004 | 0.83 |  | 0.06 ± 0.03 | 2 |
| EPA 20:5n-3 | 0.44 ± 0.02 | 8.4 |  | 0.805 ± 0.002 | 11.0 |  | <0.01 | 0.13 |
| DHA 22:6n-3 | 1.49 ± 0.08 | 28 |  | 2.315 ± 0.008 | 31.9 |  | <0.01 | <0.01 |
| Sum EPA+DHA | 1.9 ± 0.1 | 37 |  | 3.12 ± 0.01 | 42.8 |  | <0.01 | <0.01 |
| Sum n-3 | 2.1 ± 0.1 | 39 |  | 3.31 ± 0.01 | 45.4 |  | 0.07 ± 0.03 | 3 |
| Sum identified FAs | 5.23 ± 0.02 |  |  | 7.4 ± 0.2 |  |  | 2.66 ± 0.03 |  |
| n-6:n-3 ratio | 0.20 ± 0.02 |  |  | 0.149 ± 0.008 |  |  | 10 ± 6 |  |
| EPA:DHA ratio | 0.294 ± 0.005 |  |  | 0.3475 ± 0.0006 |  |  | * |  |
| ARA:EPA ratio | 0.150 ± 0.005 |  |  | 0.123 ± 0.002 |  |  | 2.2 ± 1.0 |  |
|  |  |  |  |  |  |  |  |  |
| Neutral lipid fraction |  |  |  |  |  |  |  |  |
| Sum SFA | 66.0 ± 0.5 | 44.2 |  | 63.25 ± 0.05 | 44.44 |  | 67.0 ± 0.5 | 44.79 |
| Sum MUFA | 54.3 ± 0.2 | 36.37 |  | 51.37 ± 0.07 | 35.95 |  | 54.5 ± 0.6 | 36.44 |
| LA 18:2n-6 | 22.02 ± 0.03 | 14.75 |  | 21.85 ± 0.02 | 15.5 |  | 22.7 ± 0.1 | 15.17 |
| ARA 20:4n-6 | 0.044 ± 0.008 | 0.029 |  | 0.192 ± 0.006 | 0.03 |  | 0.025 ± 0.005 | 0.017 |
| Sum n-6 | 22.62 ± 0.04 | 15.1 |  | 22.4222 ± 0.0004 | 15.80 |  | 23.2 ± 0.1 | 15.55 |
| ALA 18:3n-3 | 3.94 ± 0.02 | 2.639 |  | 4.04 ± 0.03 | 2.86 |  | 4.21 ± 0.02 | 2.82 |
| EPA 20:5n-3 | 0.68 ± 0.04 | 0.46 |  | 0.35 ± 0.03 | 0.24 |  | 0.064 ± 0.004 | 0.043 |
| DHA 22:6n-3 | 1.10 ± 0.07 | 0.74 |  | 0.40 ± 0.02 | 0.26 |  | <0.01 | <0.01 |
| Sum EPA+DHA | 1.8 ± 0.1 | 1.19 |  | 0.75 ± 0.05 | 0.50 |  | 0.076 ± 0.009 | 0.051 |
| Sum n-3 | 6.2 ± 0.1 | 4.18 |  | 5.26 ± 0.08 | 3.65 |  | 4.65 ± 0.05 | 3.11 |
| Sum identified FAs | 149.3 ± 0.7 |  |  | 142.5 ± 0.2 |  |  | 149 ± 1 |  |
| n-6:n-3 ratio | 3.63 ± 0.09 |  |  | 4.26 ± 0.06 |  |  | 5.00 ± 0.06 |  |
| EPA:DHA ratio | 0.617 ± 0.005 |  |  | 0.87 ± 0.03 |  |  | * |  |
| ARA:EPA ratio | 0.36 ± 0.03 |  |  | 0.56 ± 0.07 |  |  | 2.7 ± 0.2 |  |

Results are presented as mean ± SEM of three samples, with exception of the fresh cod diet that contained two samples. Results indicate mg FA in the polar and neutral lipid fractions/g Western diet. *not possible to calculate; EPA and DHA levels are under limit of quantification (<0.01 mg/g). Abbreviations: SFA; saturated fatty acids, MUFA; monounsaturated fatty acids, LA; linoleic acid, ARA; arachidonic acid, ALA; alpha-linolenic acid, EPA; eicosapentaenoic acid, DHA; docosahexaenoic acid, FAs; fatty acids.
