# Supplementary material for: Effects of Frozen Storage on Phospholipid Content in Atlantic Cod Fillets and the Influence on Diet-Induced Obesity in Mice
Source: Nutrients. 2018 May 30;10(6):695. doi: 10.3390/nu10060695 (PMC6024676; doi:10.3390/nu10060695)
Supplement: Supplementary file 1 [file nutrients-10-00695-s001.zip › Table S6. Lipid class composition in freeze dried fresh cod fillets.docx]

**Table S6**. Lipid class composition in freeze dried fresh cod fillets

|  | **Freeze-dried fresh cod fillets** | |
| --- | --- | --- |
| **Lipid class** | **mg/g** | **%** |
| PC | 27.4 ± 0.2 | 71 |
| PE | 7.0 ± 0.5 | 18.0 |
| PI | <0.01 | <0.01 |
| PS | <0.01 | <0.01 |
| LPC | 1.5 ± 0.1 | 3.7 |
| SM | 0.55 ± 0.08 | 1.4 |
| CL | <0.01 | <0.01 |
| Sum polar lipids | 36.5 ± 0.8 | 94 |
| FFA | 0.6 ± 0.4 | 1 |
| CHOL | 1.9 ± 0.7 | 5 |
| TAG | <0.01 | <0.01 |
| DAG | <0.01 | <0.01 |
| CE | <0.01 | <0.01 |
| Sum neutral lipids | 2 ± 1 | 6 |
| Sum Lipids | 39 ± 2 |  |
| Polar lipid:FFA ratio | 88 ± 61 |  |

Results are presented as mean ± SEM of three samples and indicate mg lipids/g and percent lipid class of total lipids in freeze dried fresh cod fillets. Abbreviations: PC; Phosphatidylcholine, PE; Phosphatidylethanolamine, PI; Phosphatidylinositol, PS; Phosphatidylserin, LPC; Lysophosphatidylcholine, SM; Sphingomyelin , CL; Cardiolipin, FFA; Free fatty acid, CHOL; Cholesterol, TAG; Triacylglycerol, DAG; Diacylglycerol, CE; Cholesteryl ester.
