# Supplementary material for: Effects of Frozen Storage on Phospholipid Content in Atlantic Cod Fillets and the Influence on Diet-Induced Obesity in Mice
Source: Nutrients. 2018 May 30;10(6):695. doi: 10.3390/nu10060695 (PMC6024676; doi:10.3390/nu10060695)
Supplement: Supplementary file 1 [file nutrients-10-00695-s001.zip › Table S7. Fatty acid compositions in the polar and neutral lipid fractions isolated from freeze dried fresh cod fillets.docx]

**Table S7.** Fatty acid compositions in the polar and neutral lipid fractions isolated from freeze dried fresh cod fillets

|  | **Freeze dried fresh cod fillets** | |
| --- | --- | --- |
| **Fatty acid** | **mg/g** | **%** |
| Polar lipid fraction |  |  |
| Sum SFA | 4.6 ± 0.1 | 24.3 |
| Sum MUFA | 2.51 ± 0.02 | 13.4 |
| LA 18:2n-6 | 0.245 ± 0.004 | 1.31 |
| ARA 20:4n-6 | 0.392 ± 0.007 | 2.088 |
| Sum n-6 | 0.75 ± 0.01 | 3.99 |
| ALA 18:3n-3 | 0.074 ± 0.004 | 0.40 |
| EPA 20:5n-3 | 3.2 ± 0.2 | 17.0 |
| DHA 22:6n-3 | 7.2 ± 0.1 | 38.4 |
| Sum EPA+DHA | 10.4 ± 0.2 | 55.4 |
| Sum n-3 | 10.9 ± 0.2 | 58.3 |
| Sum identified FAs | 18.8 ± 0.3 |  |
| n-6:n-3 ratio | 0.0685 ± 0.0009 |  |
| EPA:DHA ratio | 0.44 ± 0.03 |  |
| ARA:EPA ratio | 0.06 ± 0.03 |  |
|  |  |  |
| Neutral lipid fraction |  |  |
| Sum SFA | 0.9 ± 0.2 | 24 |
| Sum MUFA | 1.0 ± 0.2 | 26 |
| LA 18:2n-6 | 0.06 ± 0.01 | 1.56 |
| ARA 20:4n-6 | 0.09 ± 0.02 | 2.127 |
| Sum n-6 | 0.17 ± 0.04 | 4.13 |
| ALA 18:3n-3 | 0.025 ± 0.005 | 0.63 |
| EPA 20:5n-3 | 0.64 ± 0.09 | 16 |
| DHA 22:6n-3 | 1.2 ± 0.5 | 26 |
| Sum EPA+DHA | 1.8 ± 0.6 | 43 |
| Sum n-3 | 2.0 ± 0.6 | 46 |
| Sum identified FAs | 4 ± 1 |  |
| n-6:n-3 ratio | 0.090 ± 0.005 |  |
| EPA:DHA ratio | 0.7 ± 0.1 |  |
| ARA:EPA ratio | 0.21 ± 0.03 |  |

Results are presented as mean ± SEM of three samples and indicate mg FA/g and percent FA of sum identified FAs in the freeze dried fresh cod fillets. Abbreviations: SFA; saturated fatty acids, MUFA; monounsaturated fatty acids, LA; linoleic acid, ARA; arachidonic acid, ALA; alpha-linolenic acid, EPA; eicosapentaenoic acid, DHA; docosahexaenoic acid, FAs; fatty acids.
