# Supplementary material for: Effects of Frozen Storage on Phospholipid Content in Atlantic Cod Fillets and the Influence on Diet-Induced Obesity in Mice
Source: Nutrients. 2018 May 30;10(6):695. doi: 10.3390/nu10060695 (PMC6024676; doi:10.3390/nu10060695)
Supplement: Supplementary file 1 [file nutrients-10-00695-s001.zip › Table S8. Fatty acid compositions in the polar and neutral lipid fractions isolated from soybean oil, PL-soybean oil and cod liver oil.docx]

**Table S8.** Fatty acid compositions in the polar and neutral lipid fractions isolated from soybean oil, PL-soybean oil and cod liver oil

| **Fatty acid (mg/g)** | **Soybean oil** | **PL-soybean oil** | **Cod liver oil** |
| --- | --- | --- | --- |
| Polar lipid fraction |  |  |  |
| Sum SFA | 1.2 ± 0.1 | 75 ± 4 | 8 ± 1 |
| Sum MUFA | 2.2 ± 0.3 | 32 ± 1 | 5.2 ± 0.4 |
| LA 18:2n-6 | 4.3 ± 0.5 | 1.7 ± 0.1 | <0.01 |
| ARA 20:4n-6 | <0.01 | 1.33 ± 0.07 | <0.01 |
| Sum n-6 | 4.3 ± 0.5 | 3.6 ± 0.2 | <0.01 |
| ALA 18:3n-3 | 0.44 ± 0.05 | 0.89 ± 0.08 | <0.01 |
| EPA 20:5n-3 | <0.01 | 44 ± 5 | 1.01 ± 0.08 |
| DHA 22:6n-3 | <0.01 | 114 ± 8 | 1.4 ± 0.1 |
| Sum EPA+DHA | <0.01 | 158 ± 12 | 2.4 ± 0.2 |
| Sum n-3 | 0.45 ± 0.05 | 165 ± 13 | 2.4 ± 0.2 |
| Sum identified FAs | 8 ± 1 | 277 ± 18 | 15 ± 2 |
| n-6:n-3 ratio | 9.48 ± 0.05 | 0.022 ± 0.001 | * |
| EPA:DHA ratio | * | 0.38 ± 0.01 | 0.737 ± 0.006 |
| ARA:EPA ratio | * | 0.031 ± 0.002 | * |
|  |  |  |  |
| Neutral lipid fraction |  |  |  |
| Sum SFA | 137.8 ± 0.8 | 84.8 ± 0.6 | 147.6 ± 0.7 |
| Sum MUFA | 259 ± 2 | 163 ± 1 | 445.9 ± 1.7 |
| LA 18:2n-6 | 493 ± 3 | 262 ± 2 | 19.5 ± 0.2 |
| ARA 20:4n-6 | <0.01 | 0.34 ± 0.02 | 3.95 ± 0.04 |
| Sum n-6 | 493 ± 3 | 262 ± 2 | 28.0 ± 0.3 |
| ALA 18:3n-3 | 51.6 ± 0.5 | 28.5 ± 0.3 | 7.73 ± 0.06 |
| EPA 20:5n-3 | 0.46 ± 0.01 | 4.40 ± 0.06 | 78.6 ± 0.3 |
| DHA 22:6n-3 | <0.01 | 5.51 ± 0.09 | 113.4 ± 0.4 |
| Sum EPA+DHA | 0.46 ± 0.01 | 9.9 ± 0.2 | 192.1 ± 0.7 |
| Sum n-3 | 52.3 ± 0.5 | 41.4 ± 0.5 | 247 ± 1 |
| Sum identified FAs | 943 ± 6 | 551 ± 5 | 871 ± 4 |
| n-6:n-3 ratio | 9.43 ± 0.03 | 6.33 ± 0.02 | 0.113 ± 0.001 |
| EPA:DHA ratio | * | 0.798 ± 0.003 | 0.6931 ± 0.0004 |
| ARA:EPA ratio | * | 0.078 ± 0.006 | 0.0503 ± 0.0004 |

Results are presented as mean ± SEM of three samples and indicate mg FA/g oil. . *not possible to calculate; EPA and DHA levels are under limit of quantification (<0.01 mg/g). Abbreviations: SFA; saturated fatty acids, MUFA; monounsaturated fatty acids, LA; linoleic acid, ARA; arachidonic acid, ALA; alpha-linolenic acid, EPA; eicosapentaenoic acid, DHA; docosahexaenoic acid, FAs; fatty acids, PL; phospholipid.
