# Supplementary material for: Effects of Frozen Storage on Phospholipid Content in Atlantic Cod Fillets and the Influence on Diet-Induced Obesity in Mice
Source: Nutrients. 2018 May 30;10(6):695. doi: 10.3390/nu10060695 (PMC6024676; doi:10.3390/nu10060695)
Supplement: Supplementary file 1 [file nutrients-10-00695-s001.zip › Table S9. Compositions of the diets in experiment 2.docx]

**Table S9.** Compositions of the diets in experiment 2

| **Components (g/kg diet)** | **Fresh cod** | **Pork** | **Pork n-3 TAG** | **Pork n-3 PL** | **Low fat** |
| --- | --- | --- | --- | --- | --- |
| Freeze dried fresh cod fillets | 241.9 |  |  |  |  |
| Freeze dried pork sirloins |  | 240.6 | 240.6 | 240.6 |  |
| Casein |  |  |  |  | 217.5 |
| Corn starch | 281.6 | 288.0 | 288.0 | 288.0 |  |
| Dextrin from potato starch | 100.0 | 100.0 | 100.0 | 100.0 | 512.0 |
| Sucrose | 80.0 | 80.0 | 80.0 | 80.0 | 100.0 |
| Soybean oil | 12.0 | 12.0 | 12.0 | 2.6* |  |
| Corn oil | 8.0 | 8.0 | 8.0 | 8.0 | 70.0 |
| Milk fat | 58.2 | 56.5 | 51.3 | 53.3 |  |
| Lard | 58.2 | 56.5 | 51.3 | 53.3 |  |
| Margarine | 58.2 | 56.5 | 51.3 | 53.3 |  |
| Cod liver oil |  |  | 15.4 |  |  |
| PL-soybean oil |  |  |  | 18.7 |  |
| FAs from freeze dried cod loins | 5.5 |  |  |  |  |
| FAs from freeze dried pork sirloins |  | 10.6 | 10.6 | 10.6 |  |
| Cholesterol | 1.5 | 1.5 | 1.5 | 1.5 |  |
| Analyzed |  |  |  |  |  |
| Energy (KJ/g diet) | 20.6 ± 0.1 | 21.17 ± 0.03 | 21.17 ± 0.03 | 21.07 ± 0.08 | 18.60 ± 0.01 |

All diets were supplemented with 0.014 g/kg t-Butylhydroquinone, 35 g/kg AIN93G mineral mix, 10 g/kg AIN93VX NCR95 compliant vitamin mix, 3g/kg L-cystine, 2.5g/kg choline bitartrate and 50g/kg cellulose. The amount of fresh freeze dried fresh cod fillets, freeze dried pork sirloins and casein added is based on measurements of nitrogen in the protein powders. Crude protein concentration was calculated using the formula N*6.15 for casein and N*5.60 for cod fillets and pork. The calculated contribution of fatty acids (FAs) present in the protein sources is based on measurements of FA in the polar and neutral lipid fractions from the protein powders measured with GC-FID. Analyzed values represents mean ± SEM of three samples. *The PL-soybean oil contributed with 9.4 g soybean oil/kg diet. Hence, the final content of soybean oil in the Pork n-3 PL diet was 12.0 g/diet. Abbreviations: FAs; Fatty acids, PL; phospholipids.
